# Supplementary material for: Using Hawkes Processes to model imported and local malaria cases in near-elimination settings
Source: PLoS Comput Biol. 2021 Apr 1;17(4):e1008830. doi: 10.1371/journal.pcbi.1008830 (PMC8043404; doi:10.1371/journal.pcbi.1008830)
Supplement: S1 Text — (PDF) [file pcbi.1008830.s001.pdf]

S1 Text: Additional Methods

A Fitting Hawkes Processes

Following Rasmussen et al. [1] and Rizoiu et al. [2], the likelihood of the Hawkes process can be derived as follows. We define the history, $\mathcal{H}_t$, of the disease outbreak to be the list of infection times $t_1, t_2, ..., t_n$ up to but not including the current time t. We also define the conditional intensity function (or hazard function) in terms of time t as

$$\lambda(t) = \frac{f^\star(t)}{1 - F^\star(t)} \tag{A}$$

where $f^\star(t) := f(t|\mathcal{H}_t)$ is the conditional probability density function of the time of the next event t_{n+1} given the history of the previous events $t_1, t_2, ..., t_n$ and $F^\star(t)$ is the corresponding cumulative distribution function. Equations for $f^\star(t)$ and $F^\star(t)$ are derived as follows.

We first write the conditional intensity function, (A), just in terms of the cumulative distribution function $F^\star(t)$

$$\lambda(t) = \frac{\frac{\partial}{\partial t} F^\star(t)}{1 - F^\star(t)}. \tag{B}$$

We then simplify (B) into

$$\lambda(t) = -\frac{\partial}{\partial t} \log(1 - F^\star(T)). \tag{C}$$

If we integrate (C) between times t_n and t we get

$$\int_{t_n}^{t} \lambda(\tau)\, \mathrm{d}\tau = -[\log(1 - F^\star(t)) - \log(1 - F^\star(t_n))], \tag{D}$$

where t is the current time and t_n is the time of the last known event prior to t. Since $F^\star(t_n) = 0$, because $t_{n+1} > t_n$, (D) can be simplified to be

$$\int_{t_n}^{t} \lambda(\tau)\, \mathrm{d}\tau = -\log(1 - F^\star(t)). \tag{E}$$

Rearranging (D) gives us the equation for the cumulative distribution function

$$F^\star(t) = 1 - e^{-\int_{t_n}^{t} \lambda(\tau)\, \mathrm{d}\tau}. \tag{F}$$

Substituting (F) into (A) enables us to solve for the conditional probability density function

$$\lambda(t) = \frac{f^\star(t)}{1-(1-e^{-\int_{t_n}^{t}\lambda(\tau)\,\mathrm{d}\tau})}. \tag{G}$$

After rearranging (G),

$$f^\star(t) = \lambda(t)e^{-\int_{t_n}^{t}\lambda(\tau)\,\mathrm{d}\tau}. \tag{H}$$

The likelihood function of the Hawkes Process, with parameters θ is the joint density function of all the points in the history of the outbreak and can therefore be factorised into all the conditional densities of each points given all points before it. This yields

$$L(\theta) = f^\star(t_1)...f^\star(t_n)(1-F^\star(T)), \tag{I}$$

where $(1-F^\star(T))$ is the last term because the unobserved point t_{n+1} appears after the end of the observation interval. Using equation (A), the likelihood function can be written as

$$L(\theta) = \prod_{i=1}^{n} f^\star(t_i)\frac{f^\star(T)}{\lambda(T)}. \tag{J}$$

Expanding (J) using the equation for the conditional probability density function from (H) gives

$$L(\theta) = \prod_{i=1}^{n} \lambda(t_i)e^{-\int_{t_{i-1}}^{t_i}\lambda(\tau)\,\mathrm{d}\tau}\frac{\lambda(T)e^{-\int_{t_n}^{T}\lambda(\tau)\,\mathrm{d}\tau}}{\lambda(T)}. \tag{K}$$

Simplifying (K) yields

$$L(\theta) = \prod_{i=1}^{n} \lambda(t_i)e^{-\int_{t_{i-1}}^{t_i}\lambda(\tau)\,\mathrm{d}\tau}e^{-\int_{t_n}^{T}\lambda(\tau)\,\mathrm{d}\tau}. \tag{L}$$

By combining the exponentials in (L) and assuming $t_0 = 0$, the likelihood function is

$$L(\theta) = \prod_{i=1}^{n} \lambda(t_i)e^{-\int_{0}^{T}\lambda(\tau)\,\mathrm{d}\tau}. \tag{M}$$

One method for selecting parameters is to maximise our likelihood function over our parameter space Θ, which is defined as

$$\hat{\theta} = \mathrm{argmax}_{\theta\in\Theta}\left(L(\theta)\right). \tag{N}$$

However, it is common to minimise the negative log-likelihood function instead of maximising it because it is a less computationally expensive calculation and is more accurate. We therefore define our problem as

$$\hat{\theta} = \mathrm{argmin}_{\theta\in\Theta}\left(-\log L(\theta)\right), \tag{O}$$

where

$$\log L(\theta) = \sum_{i=1}^{n}\log\lambda(t_i) - \int_0^T \lambda(\tau)\,\mathrm{d}\tau \tag{P}$$

and n is the number of events at time T [3].

Traditionally, a monotonically decreasing exponential kernel of the form

$$\phi(t - t_i) = \alpha e^{-\delta * (t - t_i)} \quad \text{(Q)}$$

is used in the Hawkes process literature [4, 5, 6] where $\delta > \alpha > 0$. Here α controls the magnitude of the kernel, δ controls the speed of the decrease and i is an index. This kernel is traditionally chosen because in the most common use cases such as earthquakes and social media, events are most likely to trigger secondary events immediately after the first event happens. We discuss alternative kernels in the Methods Section that may be better suited to epidemiological modelling, where for example latent periods are necessary to capturing disease specific behaviour.

B Simulating Hawkes Processes

Simulation is used to learn more about our Hawkes Process so that we can better understand their behaviour and can validate our models to see how well they fit the underlying data. We can also use them to infer future behaviour. Ogata's thinning algorithm [7] is a method for simulating non-homogeneous Poisson processes for any kernel function $\phi(t)$; we describe this algorithm adapted for Hawkes Processes in Algorithm 1.

Supplementary Algorithm 1: Ogata's thinning algorithm adapted for Hawkes Processes

```
Set current time t = 0 and event counter i = 0;
while t ≤ T_max do
    (a) Calculate the upper bound of the Hawkes intensity λ* = λ(t+). If an event occurs
        at time t it is accounted for;
    (b) Sample inter-arrival time by drawing u ~ U(0,1) and letting τ = -ln u / λ;
    (c) Update current time: t = t + τ;
    (d) Draw s ~ U(0,1).;
    if If s ≤ λ(t)/λ* then
        Accept the current sample and let t_i = t and i = i + 1;
    else
        Reject the sample;
    end
end
```

While the current time is less than the maximum time considered in the simulation, we calculate the maximum value of the intensity, $\lambda^\star$, for the events that have happened. For any bounded intensity $\lambda(t)$, there is constant $\lambda^\star$ such that $\lambda(t) \leq \lambda^\star$ in a given time interval. The upper bound of intensity is immediately after the event has occurred for a Hawkes Process with a monotonically decreasing kernel function, like the exponential function in Eq Q, and no or a constant μ (exogenous term). However, this is not always so simple for other kernel functions and is addressed in the Methods Section.

Next we sample an inter-arrival time, τ; the greater the maximum intensity, the higher the chance of subsequent infection arising and the shorter the suggested arrival time. This is then used to update the current time and the new event is then accepted or rejected according to $\lambda^\star$. If the event is accepted, the inter-arrival time is recorded and the event count incremented. Otherwise, we reject the inter-arrival time and repeat the sampling until one is accepted or the maximum time of the simulation is reached. Even if an inter-arrival time is rejected, the time counter is still updated [7]. The upper bound, $\lambda^\star$ is updated even in the case of a rejected inter-arrival time to improve efficiency because of the strict monotonicity of $\lambda(t)$ in between event times.

Supplementary References

[1] Rasmussen, J. G. Bayesian Inference for Hawkes Processes. *Methodology and Computing in Applied Probability* **15**, 623–642 (2013).

[2] Rizoiu, M.-A., Lee, Y., Mishra, S. & Xie, L. *Hawkes Processes for Events in Social Media*, 191–218 (Association for Computing Machinery and Morgan & Claypool, 2017). URL `https://doi.org/10.1145/3122865.3122874`.

[3] Daley, D. J. & Vere-Jones, D. *An introduction to the theory of point processes*, vol. I (2008).

[4] Kelly, J. D. *et al.* Real-time predictions of the 2018–2019 Ebola virus disease outbreak in the Democratic Republic of the Congo using Hawkes point process models. *Epidemics* **28**, 100354 (2019). URL `http://www.sciencedirect.com/science/article/pii/S1755436519300258`.

[5] Rizoiu, M.-A. *et al.* Expecting to be HIP: Hawkes Intensity Processes for Social Media Popularity. In *World Wide Web 2017, International Conference on*, 1069–1078 (Perth, Australia, 2017). URL `http://arxiv.org/abs/1602.06033`. `1602.06033`.

[6] Embrechts, P., Liniger, T. & Lin, L. Multivariate Hawkes processes: an application to financial data. *Journal of Applied Probability* **48**, 367–378 (2011).

[7] Ogata, Y. On Lewis' simulation method for point processes. *IEEE Transactions on Information Theory* **27**, 23–31 (1981).
