# Supplementary material for: Using Hawkes Processes to model imported and local malaria cases in near-elimination settings
Source: PLoS Comput Biol. 2021 Apr 1;17(4):e1008830. doi: 10.1371/journal.pcbi.1008830 (PMC8043404; doi:10.1371/journal.pcbi.1008830)
Supplement: S2 Text — (PDF) [file pcbi.1008830.s002.pdf]

S2 Text: Directional derivatives of the negative log-likelihood

Our fitting process is defined as

$$\hat{\theta} = \text{argmin}_{\theta \in \Theta} \left(-\log L(\theta) \right), \tag{A}$$

for

$$\theta = \{\alpha, \delta, A, B, M, N\} \tag{B}$$

where

$$\log L(\theta) = \sum_{i=1}^{n} \log \left(\mu(t_i) + \sum_{t_i > (t_j + \Delta)} \phi\left(t_i - (t_j + \Delta)\right) \right) - \int_0^{T_{\max}} \mu(\tau)\,\mathrm{d}\tau - \sum_{i=1}^{n} \int_0^{T_{\max}} \phi(\tau - (t_i + \Delta))\,\mathrm{d}\tau, \tag{C}$$

and $\phi(\cdot)$ is the Rayleigh kernel in equation 6 of the paper and $\mu(t)$ is given in equation 9 of the paper. We define the integral of μ with respects to t between 0 and $T_{\max}$ to be

$$\int_0^{T_{\max}} \mu(t)\,\mathrm{d}t = \int_T A + Bt + M\cos\left(\frac{2\pi t}{p}\right) + N\sin\left(\frac{2\pi t}{p}\right)\mathrm{d}t, \tag{D}$$

where

$$T = t : A + Bt + M\cos\left(\frac{2\pi t}{p}\right) + N\sin\left(\frac{2\pi t}{p}\right) > 0. \tag{E}$$

Assuming that there are a finite number of regions between 0 and $T_{\max}$ where $A + Bt + M\cos\left(\frac{2\pi t}{p}\right) + N\sin\left(\frac{2\pi t}{p}\right) > 0$ and each region is between $[a_k, b_{kj}]$,

$$\int_0^{T_{\max}} \mu(t)\,\mathrm{d}t = \sum_k \int_{a_k}^{b_k} A + Bt + M\cos\left(\frac{2\pi t}{p}\right) + N\sin\left(\frac{2\pi t}{p}\right)\mathrm{d}t \tag{F}$$

$$= \sum_k \left[At + \frac{Bt^2}{2} + \frac{Mp}{2\pi}\sin\left(\frac{2\pi t}{p}\right) - \frac{Np}{2\pi}\cos\left(\frac{2\pi t}{p}\right) \right]_{a_k}^{b_k}. \tag{G}$$

Therefore, in full

$$\log L(\theta) = \sum_{i=1}^{n} \log \left(\max \left(A + Bt_i + M \cos\left(\frac{2\pi t_i}{p}\right) + N \sin\left(\frac{2\pi t_i}{p}\right), 0 \right) + \sum_{t_i > (t_j + \Delta)} \alpha * (t_i - (t_j + \Delta)) e^{-\delta * (t_i - (t_j + \Delta))^2/2} \right) - \sum_k \left[At + \frac{Bt^2}{2} + \frac{Mp}{2\pi} \sin\left(\frac{2\pi t}{p}\right) - \frac{Np}{2\pi} \cos\left(\frac{2\pi t}{p}\right) \right]_{a_k}^{b_k} - \sum_{i=1}^{n} \frac{\alpha}{\delta} \left(1 - e^{-\delta(T_{\max} - (t_i + \Delta))^2/2} \right). \tag{H}$$

We need directional derivatives of the likelihood with respect to each parameter as an input for the optimisation algorithm. These are written out below for completeness. We define

$$\zeta_i = \max \left(A + Bt_i + M \cos\left(\frac{2\pi t_i}{p}\right) + N \sin\left(\frac{2\pi t_i}{p}\right), 0 \right) + \sum_{t_i > (t_j + \Delta)} \alpha * (t_i - (t_j + \Delta)) e^{-\delta * (t_i - (t_j + \Delta))^2/2}. \tag{I}$$

First, we consider the directional derivatives for the kernel parameters:

$$\frac{\partial \log L(\theta)}{\partial \alpha} = \sum_{i=1}^{n} \frac{\sum_{t_i > (t_j + \Delta)} (t_i - (t_j + \Delta)) e^{-\delta * (t_i - (t_j + \Delta))^2/2}}{\zeta_i} - \sum_{i=1}^{n} \frac{1}{\delta} \left(1 - e^{-\delta * (T_{\max} - (t_i + \Delta))^2/2} \right), \tag{J}$$

$$\frac{\partial \log L(\theta)}{\partial \delta} = \sum_{i=1}^{n} \frac{\sum_{t_i > (t_j + \Delta)} -\alpha * (t - (t_j + \Delta))^3 e^{-\delta * (t_i - (t_j + \Delta))^2/2}}{2\zeta_i} + \sum_{i=1}^{n} \frac{\alpha}{\delta^2} \left(1 - e^{-\delta * (T_{\max} - (t_i + \Delta))^2/2} \left(\frac{\delta}{2} (T_{\max} - (t_i + \Delta))^2 + 1 \right) \right). \tag{K}$$

Then we consider the directional derivatives for the μ parameters; the first term of each directional derivatives is only evaluated at times, t_i, when $\mu(t_i) > 0$:

$$\frac{\partial \log L(\theta)}{\partial A} = \sum_{i=1}^{n} \frac{1}{\zeta_i} \Big|_{\mu(t_i) > 0} - \sum_k [t]_{a_k}^{b_k}, \tag{L}$$

$$\frac{\partial \log L(\theta)}{\partial B} = \sum_{i=1}^{n} \frac{t_i}{\zeta_i} \Big|_{\mu(t_i) > 0} - \sum_k \left[\frac{t^2}{2} \right]_{a_k}^{b_k}, \tag{M}$$

$$\frac{\partial \log L(\theta)}{\partial M} = \sum_{i=1}^{n} \frac{\cos\left(\frac{2\pi t_i}{p}\right)}{\zeta_i} \Big|_{\mu(t_i) > 0} - \sum_k \left[\frac{p}{2\pi} \sin\left(\frac{2\pi t}{p}\right) \right]_{a_k}^{b_k}, \tag{N}$$

$$\frac{\partial \log L(\theta)}{\partial N} = \sum_{i=1}^{n} \frac{\sin\left(\frac{2\pi t_i}{p}\right)}{\zeta_i} \Big|_{\mu(t_i) > 0} + \sum_k \left[\frac{p}{2\pi} \cos\left(\frac{2\pi t}{p}\right) \right]_{a_k}^{b_k}. \tag{O}$$

The derivative of μ with respects to t is required for the simulation so included here for completeness

$$\frac{\mathrm{d}\mu}{\mathrm{d}t} = \max\left(Bt - \frac{2\pi M}{p}\sin\left(\frac{2\pi t}{p}\right) + \frac{2\pi N}{p}\cos\left(\frac{2\pi t}{p}\right), 0\right). \tag{P}$$

We also provide an analytic solution for our hessian. First we consider the directional derivatives of ζ_i.

$$\frac{\partial \zeta_i}{\partial \alpha} = \sum_{t_i > (t_j+\Delta)} (t_i - (t_j+\Delta)) e^{-\delta*(t_i-(t_j+\Delta))^2/2}, \tag{Q}$$

$$\frac{\partial \zeta_i}{\partial \delta} = -\sum_{t_i > (t_j+\Delta)} \frac{\alpha}{2} * (t_i - (t_j+\Delta))^3 e^{-\delta*(t_i-(t_j+\Delta))^2/2}, \tag{R}$$

$$\frac{\partial \zeta_i}{\partial A} = 1\Big|_{\mu(t_i)>0}, \tag{S}$$

$$\frac{\partial \zeta_i}{\partial B} = t_i\Big|_{\mu(t_i)>0}, \tag{T}$$

$$\frac{\partial \zeta_i}{\partial M} = \cos\left(\frac{2\pi t_i}{p}\right)\Big|_{\mu(t_i)>0}, \tag{U}$$

$$\frac{\partial \zeta_i}{\partial N} = \sin\left(\frac{2\pi t_i}{p}\right)\Big|_{\mu(t_i)>0}. \tag{V}$$

The constituent parts of the hessian are therefore:

$$\frac{\partial^2 \log L(\theta)}{\partial \alpha^2} = -\sum_{i=1}^{n} \frac{\sum_{t_i>(t_j+\Delta)}(t_i-(t_j+\Delta))e^{-\delta*(t_i-(t_j+\Delta))^2/2}}{\zeta_i^2}\frac{\partial \zeta_i}{\partial \alpha}, \tag{W}$$

$$\begin{aligned}\frac{\partial^2 \log L(\theta)}{\partial \alpha \partial \delta} = \sum_{i=1}^{n}\Bigg[&-\frac{\sum_{t_i>(t_j+\Delta)}(t_i-(t_j+\Delta))e^{-\delta*(t_i-(t_j+\Delta))^2/2}}{\zeta_i}\left(\frac{(t_i-(t_j+\Delta))^2}{2} + \frac{1}{\zeta_i}\frac{\partial \zeta_i}{\partial \delta}\right) \\ &-\frac{e^{-\delta*(t_i-(t_j+\Delta))^2/2}}{\delta}\left(\frac{(T_{\max}-(t_i+\Delta))^2}{2} + \frac{1}{\delta}\right) + \frac{1}{\delta^2}\Bigg],\end{aligned} \tag{X}$$

$$\frac{\partial^2 \log L(\theta)}{\partial \alpha \partial A} = -\sum_{i=1}^{n} \frac{\sum_{t_i>(t_j+\Delta)}(t_i-(t_j+\Delta))e^{-\delta*(t_i-(t_j+\Delta))^2/2}}{\zeta_i^2}\frac{\partial \zeta_i}{\partial A}, \tag{Y}$$

$$\frac{\partial^2 \log L(\theta)}{\partial \alpha \partial B} = -\sum_{i=1}^{n} \frac{\sum_{t_i>(t_j+\Delta)}(t_i-(t_j+\Delta))e^{-\delta*(t_i-(t_j+\Delta))^2/2}}{\zeta_i^2}\frac{\partial \zeta_i}{\partial B}, \tag{Z}$$

$$\frac{\partial^2 \log L(\theta)}{\partial \alpha \partial M} = -\sum_{i=1}^{n} \frac{\sum_{t_i>(t_j+\Delta)}(t_i-(t_j+\Delta))e^{-\delta*(t_i-(t_j+\Delta))^2/2}}{\zeta_i^2}\frac{\partial \zeta_i}{\partial M}, \tag{AA}$$

$$\frac{\partial^2 \log L(\theta)}{\partial \alpha \partial N} = -\sum_{i=1}^{n} \frac{\sum_{t_i>(t_j+\Delta)}(t_i-(t_j+\Delta))e^{-\delta*(t_i-(t_j+\Delta))^2/2}}{\zeta_i^2}\frac{\partial \zeta_i}{\partial N}, \tag{AB}$$

$$\frac{\partial^2 \log L(\theta)}{\partial\delta\partial\alpha} = \frac{1}{\alpha}\frac{\partial \log L(\theta)}{\partial\alpha} + \sum_{i=1}^{n} \frac{\alpha}{2} \frac{\sum_{t_i>(t_j+\Delta)}(t_i-(t_j+\Delta))^3 e^{-\delta*(t_i-(t_j+\Delta))^2/2}}{\zeta_i^2}\frac{\partial\zeta_i}{\partial\alpha}, \tag{AC}$$

$$\begin{aligned}\frac{\partial^2 \log L(\theta)}{\partial\delta^2} = \sum_{i=1}^{n} \alpha \Bigg[& \frac{\sum_{t_i>(t_j+\Delta)}(t_i-(t_j+\Delta))^3 e^{-\delta*(t_i-(t_j+\Delta))^2/2}}{2\zeta_i}\left(\frac{(t_i-(t_j+\Delta))^2}{2}+\frac{1}{\zeta_i}\frac{\partial\zeta_i}{\partial\delta}\right) \\ & -\frac{2}{\delta^3}\left(1-e^{-\delta*(T_{\max}-(t_i+\Delta))^2/2}\right) \\ & +\frac{(T_{\max}-(t_i+\Delta))^2 e^{-\delta*(T_{\max}-(t_i+\Delta))^2/2}}{\delta^2}\left(1+\frac{\delta(T_{\max}-(t_i+\Delta))^2}{4}\right)\Bigg],\end{aligned} \tag{AD}$$

$$\frac{\partial^2 \log L(\theta)}{\partial\delta\partial A} = \sum_{i=1}^{n} \frac{\alpha}{2} \frac{\sum_{t_i>(t_j+\Delta)}(t_i-(t_j+\Delta))^3 e^{-\delta*(t_i-(t_j+\Delta))^2/2}}{\zeta_i^2}\frac{\partial\zeta_i}{\partial A}, \tag{AE}$$

$$\frac{\partial^2 \log L(\theta)}{\partial\delta\partial B} = \sum_{i=1}^{n} \frac{\alpha}{2} \frac{\sum_{t_i>(t_j+\Delta)}(t_i-(t_j+\Delta))^3 e^{-\delta*(t_i-(t_j+\Delta))^2/2}}{\zeta_i^2}\frac{\partial\zeta_i}{\partial B}, \tag{AF}$$

$$\frac{\partial^2 \log L(\theta)}{\partial\delta\partial M} = \sum_{i=1}^{n} \frac{\alpha}{2} \frac{\sum_{t_i>(t_j+\Delta)}(t_i-(t_j+\Delta))^3 e^{-\delta*(t_i-(t_j+\Delta))^2/2}}{\zeta_i^2}\frac{\partial\zeta_i}{\partial M}, \tag{AG}$$

$$\frac{\partial^2 \log L(\theta)}{\partial\delta\partial N} = \sum_{i=1}^{n} \frac{\alpha}{2} \frac{\sum_{t_i>(t_j+\Delta)}(t_i-(t_j+\Delta))^3 e^{-\delta*(t_i-(t_j+\Delta))^2/2}}{\zeta_i^2}\frac{\partial\zeta_i}{\partial N}, \tag{AH}$$

$$\frac{\partial^2 \log L(\theta)}{\partial A\partial\alpha} = -\sum_{i=1}^{n} \frac{1}{\zeta_i^2}\frac{\partial\zeta_i}{\partial\alpha}\Big|_{\mu(t_i)>0}, \tag{AI}$$

$$\frac{\partial^2 \log L(\theta)}{\partial A\partial\delta} = -\sum_{i=1}^{n} \frac{1}{\zeta_i^2}\frac{\partial\zeta_i}{\partial\delta}\Big|_{\mu(t_i)>0}, \tag{AJ}$$

$$\frac{\partial^2 \log L(\theta)}{\partial A^2} = -\sum_{i=1}^{n} \frac{1}{\zeta_i^2}\frac{\partial\zeta_i}{\partial A}\Big|_{\mu(t_i)>0}, \tag{AK}$$

$$\frac{\partial^2 \log L(\theta)}{\partial A\partial B} = -\sum_{i=1}^{n} \frac{1}{\zeta_i^2}\frac{\partial\zeta_i}{\partial B}\Big|_{\mu(t_i)>0}, \tag{AL}$$

$$\frac{\partial^2 \log L(\theta)}{\partial A\partial M} = -\sum_{i=1}^{n} \frac{1}{\zeta_i^2}\frac{\partial\zeta_i}{\partial M}\Big|_{\mu(t_i)>0}, \tag{AM}$$

$$\frac{\partial^2 \log L(\theta)}{\partial A\partial N} = -\sum_{i=1}^{n} \frac{1}{\zeta_i^2}\frac{\partial\zeta_i}{\partial N}\Big|_{\mu(t_i)>0}, \tag{AN}$$

$$\frac{\partial^2 \log L(\theta)}{\partial B\partial\alpha} = -\sum_{i=1}^{n} \frac{t_i}{\zeta_i^2}\frac{\partial\zeta_i}{\partial\alpha}\Big|_{\mu(t_i)>0}, \tag{AO}$$

$$\frac{\partial^2 \log L(\theta)}{\partial B \partial \delta} = -\sum_{i=1}^{n} \frac{t_i}{\zeta_i^2} \frac{\partial \zeta_i}{\partial \delta}\Big|_{\mu(t_i)>0}, \tag{AP}$$

$$\frac{\partial^2 \log L(\theta)}{\partial B \partial A} = -\sum_{i=1}^{n} \frac{t_i}{\zeta_i^2} \frac{\partial \zeta_i}{\partial A}\Big|_{\mu(t_i)>0}, \tag{AQ}$$

$$\frac{\partial^2 \log L(\theta)}{\partial B^2} = -\sum_{i=1}^{n} \frac{t_i}{\zeta_i^2} \frac{\partial \zeta_i}{\partial B}\Big|_{\mu(t_i)>0}, \tag{AR}$$

$$\frac{\partial^2 \log L(\theta)}{\partial B \partial M} = -\sum_{i=1}^{n} \frac{t_i}{\zeta_i^2} \frac{\partial \zeta_i}{\partial M}\Big|_{\mu(t_i)>0}, \tag{AS}$$

$$\frac{\partial^2 \log L(\theta)}{\partial B \partial N} = -\sum_{i=1}^{n} \frac{t_i}{\zeta_i^2} \frac{\partial \zeta_i}{\partial N}\Big|_{\mu(t_i)>0}, \tag{AT}$$

$$\frac{\partial^2 \log L(\theta)}{\partial M \partial \alpha} = -\sum_{i=1}^{n} \frac{\cos\left(\frac{2\pi t_i}{p}\right)}{\zeta_i^2} \frac{\partial \zeta_i}{\partial \alpha}\Big|_{\mu(t_i)>0}, \tag{AU}$$

$$\frac{\partial^2 \log L(\theta)}{\partial M \partial \delta} = -\sum_{i=1}^{n} \frac{\cos\left(\frac{2\pi t_i}{p}\right)}{\zeta_i^2} \frac{\partial \zeta_i}{\partial \delta}\Big|_{\mu(t_i)>0}, \tag{AV}$$

$$\frac{\partial^2 \log L(\theta)}{\partial M \partial A} = -\sum_{i=1}^{n} \frac{\cos\left(\frac{2\pi t_i}{p}\right)}{\zeta_i^2} \frac{\partial \zeta_i}{\partial A}\Big|_{\mu(t_i)>0}, \tag{AW}$$

$$\frac{\partial^2 \log L(\theta)}{\partial M \partial B} = -\sum_{i=1}^{n} \frac{\cos\left(\frac{2\pi t_i}{p}\right)}{\zeta_i^2} \frac{\partial \zeta_i}{\partial B}\Big|_{\mu(t_i)>0}, \tag{AX}$$

$$\frac{\partial^2 \log L(\theta)}{\partial M^2} = -\sum_{i=1}^{n} \frac{\cos\left(\frac{2\pi t_i}{p}\right)}{\zeta_i^2} \frac{\partial \zeta_i}{\partial M}\Big|_{\mu(t_i)>0}, \tag{AY}$$

$$\frac{\partial^2 \log L(\theta)}{\partial M \partial N} = -\sum_{i=1}^{n} \frac{\cos\left(\frac{2\pi t_i}{p}\right)}{\zeta_i^2} \frac{\partial \zeta_i}{\partial N}\Big|_{\mu(t_i)>0}, \tag{AZ}$$

$$\frac{\partial^2 \log L(\theta)}{\partial N \partial \alpha} = -\sum_{i=1}^{n} \frac{\sin\left(\frac{2\pi t_i}{p}\right)}{\zeta_i^2} \frac{\partial \zeta_i}{\partial \alpha}\Big|_{\mu(t_i)>0}, \tag{BA}$$

$$\frac{\partial^2 \log L(\theta)}{\partial N \partial \delta} = -\sum_{i=1}^{n} \frac{\sin\left(\frac{2\pi t_i}{p}\right)}{\zeta_i^2} \frac{\partial \zeta_i}{\partial \delta}\Big|_{\mu(t_i)>0}, \tag{BB}$$

$$\frac{\partial^2 \log L(\theta)}{\partial N \partial A} = -\sum_{i=1}^{n} \frac{\sin\left(\frac{2\pi t_i}{p}\right)}{\zeta_i^2} \frac{\partial \zeta_i}{\partial A}\Big|_{\mu(t_i)>0}, \tag{BC}$$

$$\frac{\partial^2 \log L(\theta)}{\partial N \partial B} = -\sum_{i=1}^{n} \frac{\sin\left(\frac{2\pi t_i}{p}\right)}{\zeta_i^2} \frac{\partial \zeta_i}{\partial B}\Big|_{\mu(t_i)>0}, \tag{BD}$$

$$\frac{\partial^2 \log L(\theta)}{\partial N \partial M} = -\sum_{i=1}^{n} \frac{\sin\left(\frac{2\pi t_i}{p}\right)}{\zeta_i^2} \frac{\partial \zeta_i}{\partial M}\Big|_{\mu(t_i)>0}, \tag{BE}$$

$$\frac{\partial^2 \log L(\theta)}{\partial N^2} = -\sum_{i=1}^{n} \frac{\sin\left(\frac{2\pi t_i}{p}\right)}{\zeta_i^2} \frac{\partial \zeta_i}{\partial N}\Big|_{\mu(t_i)>0}. \tag{BF}$$
