# Supplementary material for: Using Hawkes Processes to model imported and local malaria cases in near-elimination settings
Source: PLoS Comput Biol. 2021 Apr 1;17(4):e1008830. doi: 10.1371/journal.pcbi.1008830 (PMC8043404; doi:10.1371/journal.pcbi.1008830)
Supplement: S3 Text — (PDF) [file pcbi.1008830.s003.pdf]

S3 Text: Maximum intensity for a Rayleigh kernel

There is no analytic solution for the time of the maximum value of the intensity function, $\lambda_{\max}$, after the last event or infection so it is no longer trivial to find the maximum value of the intensity. We are however able to bound the region of time in which this maximum lies by considering the superposition of multiple Rayleigh kernels and the functional form of the exogenous term μ. We consider $\mu = 0$ in this appendix and present the results when μ varies in thee methods section. First we assume there is no delay in a person becoming infectious once they have been infected and then consider a delay.

A Rayleigh kernel without a delay

The value of the intensity function when $\mu = 0$, or is constant, can be found by superposing the Rayleigh kernels that result from individuals being infected at different times. Fig A shows examples of the intensity functions for events at different times with kernel parameters $\alpha = 1.0$ and $\delta = 1.0$. The time of the maximum value of the intensity for a single infection, $t_{\text{max intensity}}$, is known see panel A. However, as shown in panels B and C, $t_{\text{max intensity}}$ from two infectious does not occur at the same time as the maximum of either individual infection, so we can only place a bound around the time of $\lambda_{\max}$. The maximum value of the intensity after an event must occur after the last event by definition, so this forms the lower bound on the time region in which the maximum lies. Since we assume the parameters of the kernels are constant with time, all the kernels must decrease after the maximum value of the last event so the upper bound must be the maximum time of a Rayleigh kernel that starts at the time of the last event. Therefore,

$$t_{\text{last event}} < t_{\text{max intensity}} < t_{\text{last event}} + \frac{1}{\sqrt{\delta}}. \tag{A}$$

This is shown in panels B and C for two events because the solid black line is between the blue dot and dashed blue line and again shown in panel D for 6 events since the black line lies between the dark red dot and dark red dashed line.

Now we have the bound in which $t_{\text{max intensity}}$ lies, it is possible to use a root finding algorithm to locate $t_{\text{max intensity}}$ and thus $\lambda_{\max}$. We know $\lambda_{\max}$ is a turning point in $\lambda(t)$, so $t_{\text{max intensity}}$ is the root of the derivative of $\lambda_{\max}$. This is analytically

$$\begin{aligned}\frac{\mathrm{d}\lambda(t)}{\mathrm{d}t} &= \sum_{t-t_i} \alpha e^{-\delta(t-t_i)^2/2} - \alpha\delta(t-t_i)^2 e^{-\delta(t-t_i)^2/2} \\ &= \sum_{t-t_i} (1-\delta(t-t_i)^2)\alpha e^{-\delta(t-t_i)^2/2}. \end{aligned} \tag{B}$$

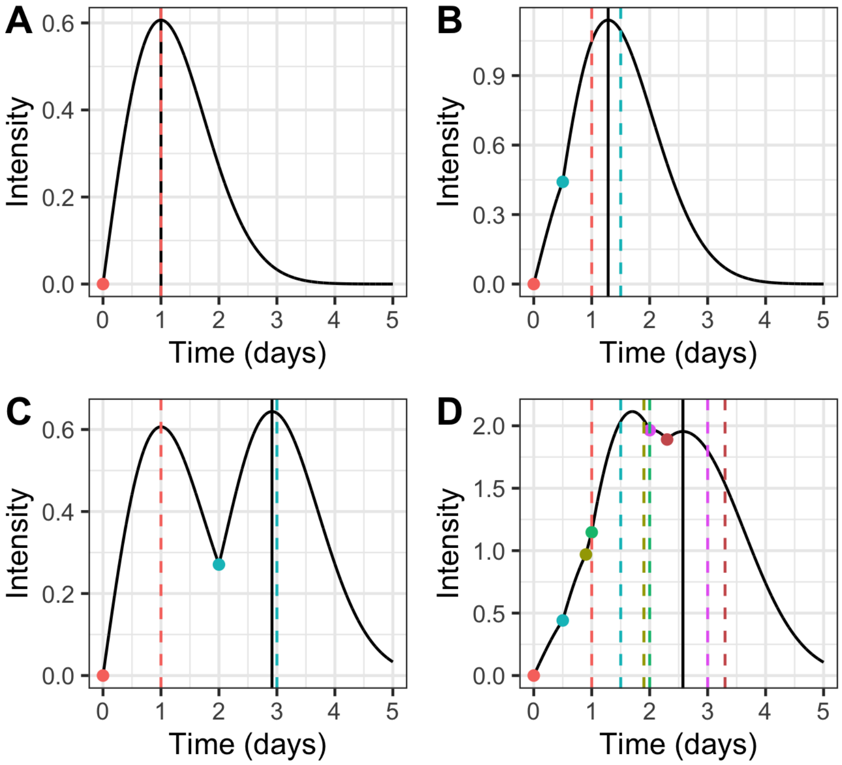

Fig A: Intensity function for events at different times with kernel parameters $\alpha = 1.0$ and $\delta = 1.0$. The coloured dots refer to different infections and the corresponding coloured dashed lines indicate the time of the theoretical maximum value of a single Rayleigh kernel at each event time. The solid black line indicates the time of the maximum value of the kernel after the last event, which results from the summing the contribution from the Rayleigh kernel from each event. Panel A shows a single Rayleigh kernel from one infection, panels B and C show the superposition of two infections and panel D shows the superposition of 6 infections.

B Rayleigh kernel with a delay

When we add a delay, there is no longer necessarily only one root between $t_{\text{last event}}$ and $t_{\text{last event}} + \frac{1}{\sqrt{\delta}} + \tau$, where τ is the delay. Therefore, using root finding algorithms for the derivative may only locate a local maximum and not the true global maximum of the intensity, which is needed for the simulation algorithm. Fig B shows examples of the intensity function for the same scenarios as Fig A, but includes a 1 day delay before a person becomes infectious. Again, in all panels

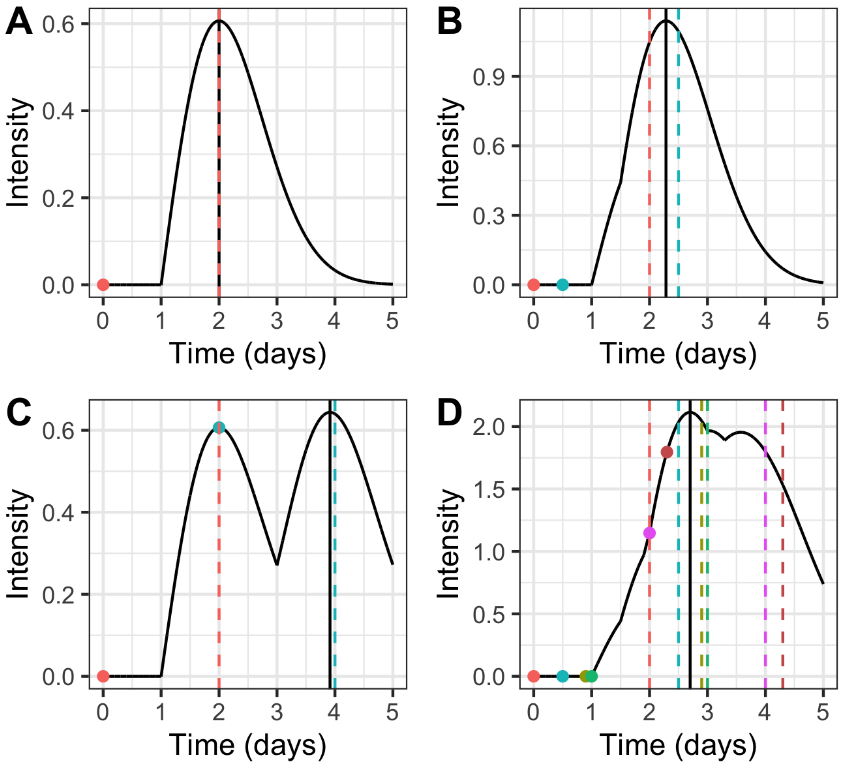

Fig B: Intensity function for events at different times with kernel parameters $\alpha = 1.0$ and $\delta = 1.0$ and a 1 day delay. The coloured dots refer to different events or infections and the corresponding coloured dashed lines indicate the time of the theoretical maximum value of a single Rayleigh kernel at each event time. The solid black line indicates the time of the maximum value of the kernel after the last event. Panel A shows a single Rayleigh kernel, panels B and C show the superposition of two events and panel D shows the superposition of 6 events.

the maximum value of the kernel lies between the time of the last event and the time of the maximum value of a single kernel at that time but in panel D it is obvious that there are three local maximum in that range so care needs to be taken to select the true maximum. This can be done by evaluating all the roots of the derivative of $\lambda(t)$ between $t_{\text{last event}}$ and $t_{\text{last event}} + \frac{1}{\sqrt{\delta}} + \tau$ and then selecting the maximum value.
