# Supplementary material for: Using Hawkes Processes to model imported and local malaria cases in near-elimination settings
Source: PLoS Comput Biol. 2021 Apr 1;17(4):e1008830. doi: 10.1371/journal.pcbi.1008830 (PMC8043404; doi:10.1371/journal.pcbi.1008830)
Supplement: S4 Text — Derivation of the branching factor for a Rayleigh kernel. (PDF) [file pcbi.1008830.s004.pdf]

S4 Text: Branching factor derivation

The branching factor, $n^\star$, is defined as the expected number of events that are directly triggered by an event in a Hawkes process. It is defined as

$$n^\star = \int_0^\infty \phi(\tau)\,\mathrm{d}\tau. \tag{A}$$

Substituting our intensity kernel into (A), and assuming $\tau = t - t_i$ results in

$$\begin{aligned} n^\star &= \int_0^\infty \alpha\tau e^{-\frac{\delta\tau^2}{2}}\,\mathrm{d}\tau, \\ &= \left[-\frac{\alpha}{\delta}e^{-\frac{\delta\tau^2}{2}}\right]_0^\infty, \\ &= \frac{\alpha}{\delta}. \end{aligned} \tag{B}$$
