# Supplementary figures and images for: Using Hawkes Processes to model imported and local malaria cases in near-elimination settings
Source: PLoS Comput Biol. 2021 Apr 1;17(4):e1008830. doi: 10.1371/journal.pcbi.1008830 (PMC8043404; doi:10.1371/journal.pcbi.1008830)

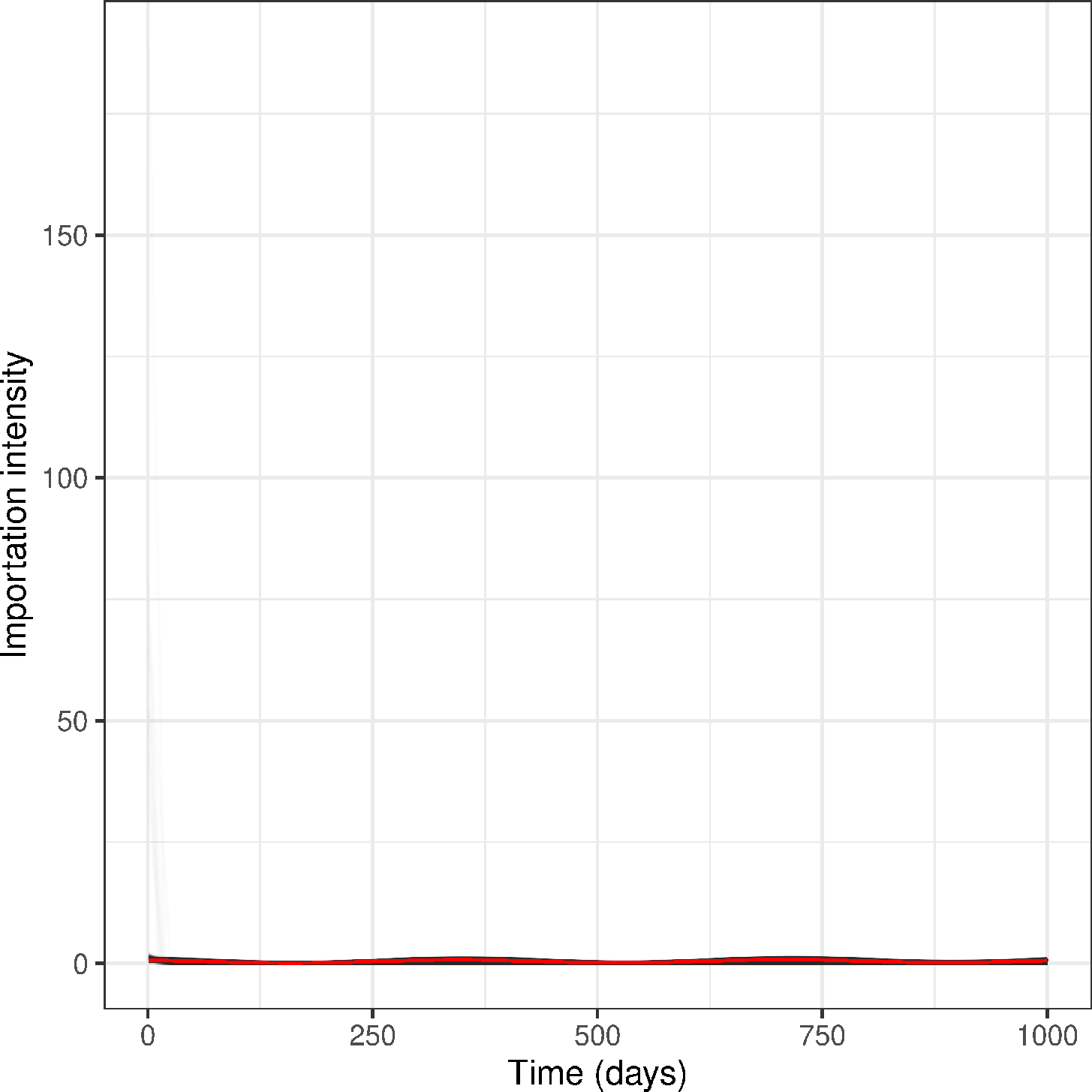

Supplement: S1 Fig — This is an un-magnified version of Fig 2B. The red line shows the importation intensity calculated from the initial parameters and the black lines shows the importation intensity calculated from the parameters fit from each simulation. (TIF) [file pcbi.1008830.s005.tif]

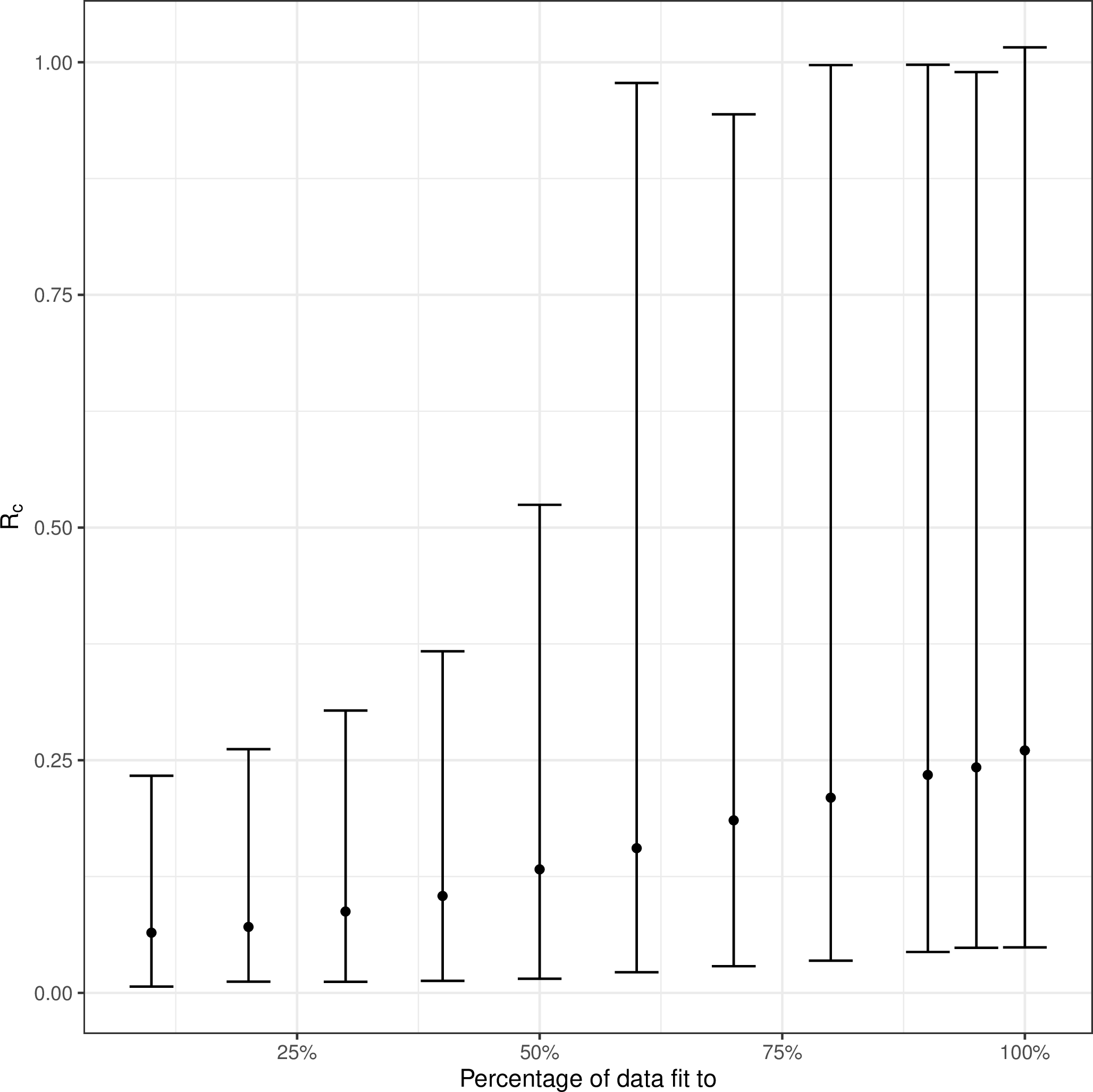

Supplement: S2 Fig — The points show our median estimate for Rc at each percentage of data fit to and the error bars show the 95% confidence intervals. (TIF) [file pcbi.1008830.s006.tif]

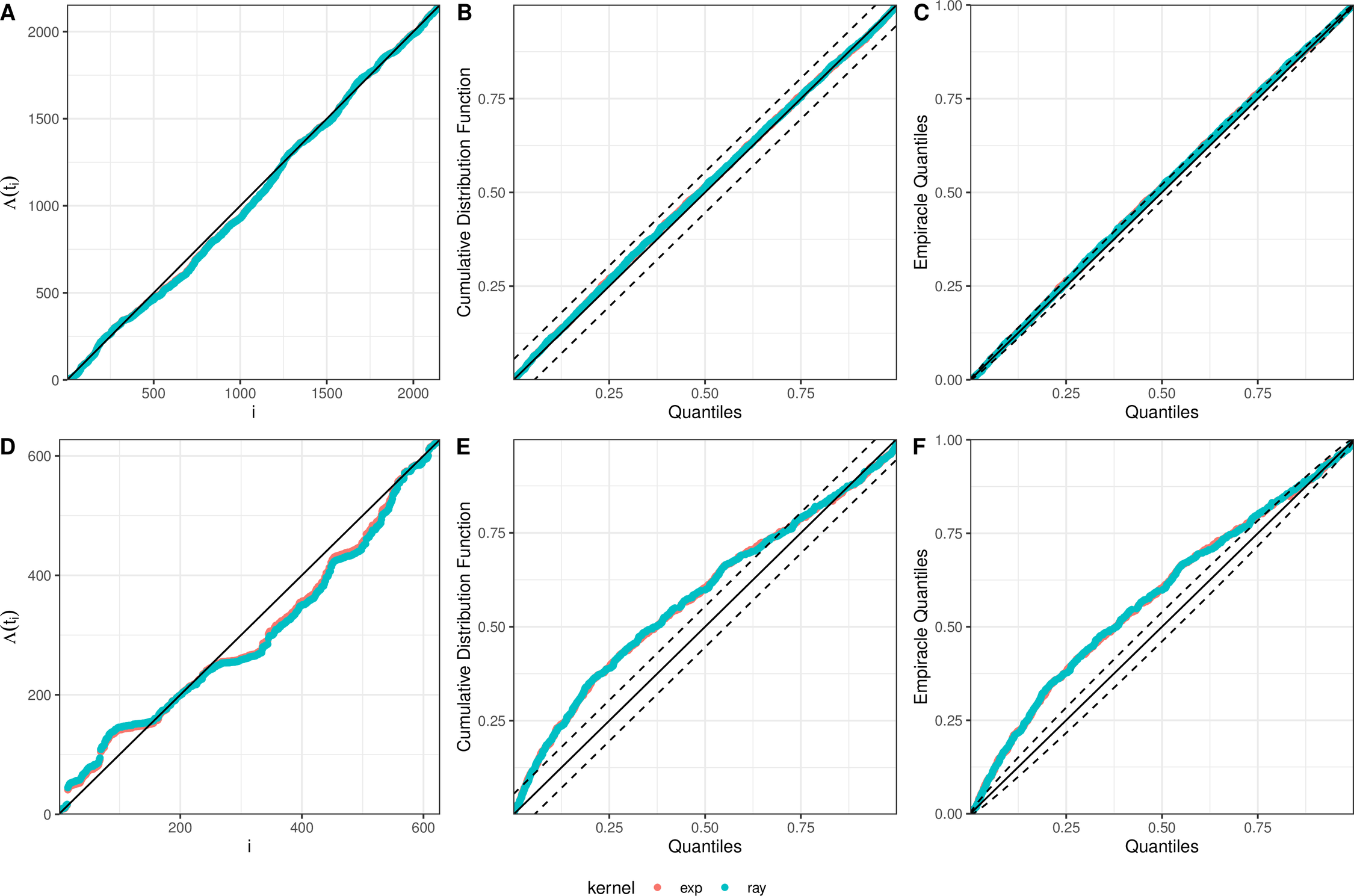

Supplement: S3 Fig — S3A and S3D Fig show Λ(ti) against i for China and Eswatini respectively, S3B and S3E Fig show Kolmogorov–Smirnov tests for China and Eswatini respectively and S3C and S3F Fig show quantile–quantile plots for China and Eswatini respectively. The solid line shows the line y = x and the dashed lines show the 95% credible intervals for each test. (TIF) [file pcbi.1008830.s007.tif]

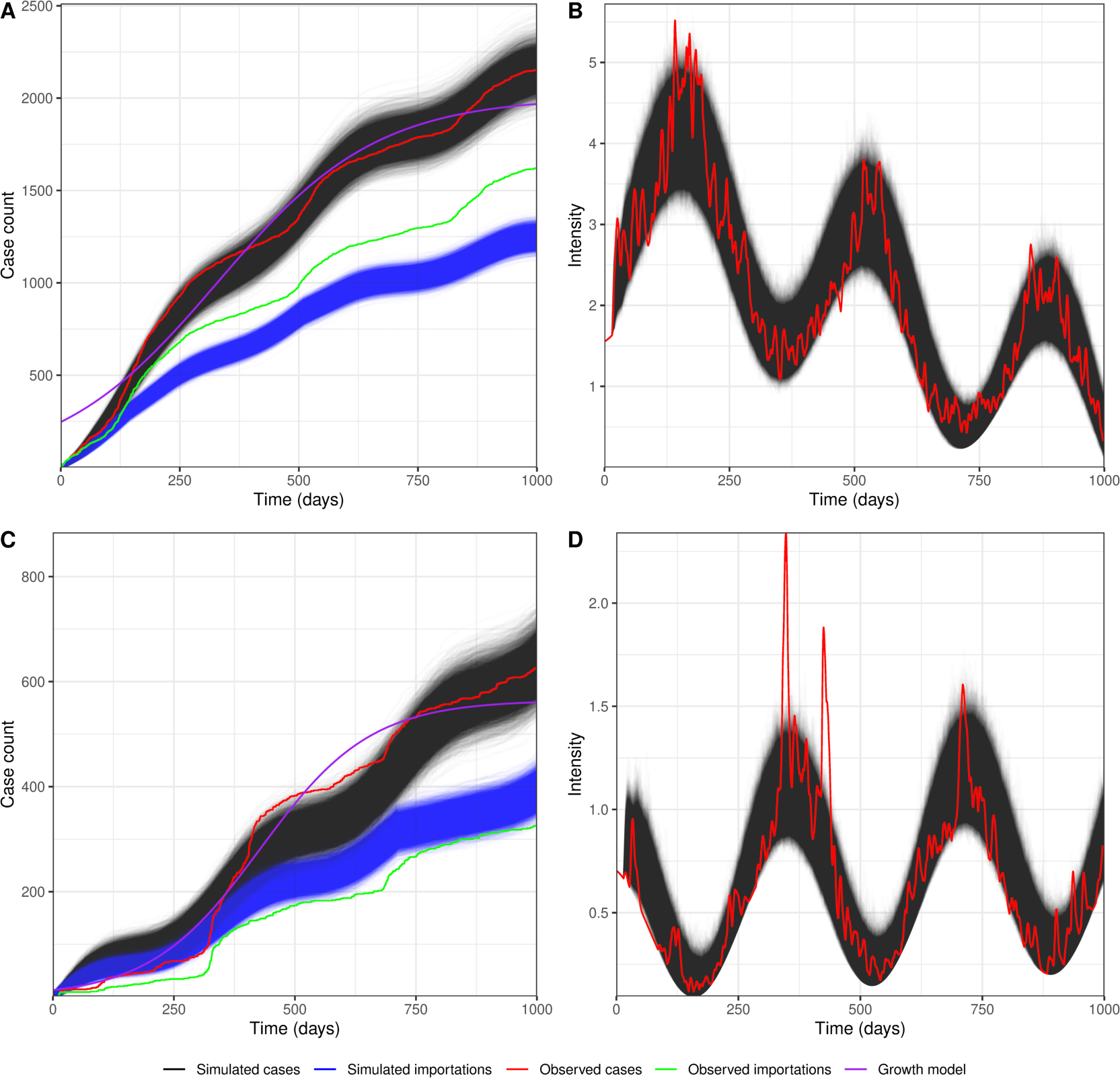

Supplement: S4 Fig — S4A and S4C Fig show malaria case counts for China and Eswatini respectively. The red line shows the real case counts over time and the black lines show the case counts over time from 10,000 simulations of the full fitted model. The green line shows the real case count over time from the cases labelled as importations and the blue lines show the case counts over time from 10,000 simulations of just the exogenous term (Eq (6)). S4B and S4D Fig shows the calculated Hawkes intensity (Eq (1)) for China and Eswatini respectively. The red line shows the intensity calculated from the fitted parameters and real events, whereas the black lines show the intensity calculated from the fitted parameters and the simulated events. (TIF) [file pcbi.1008830.s008.tif]

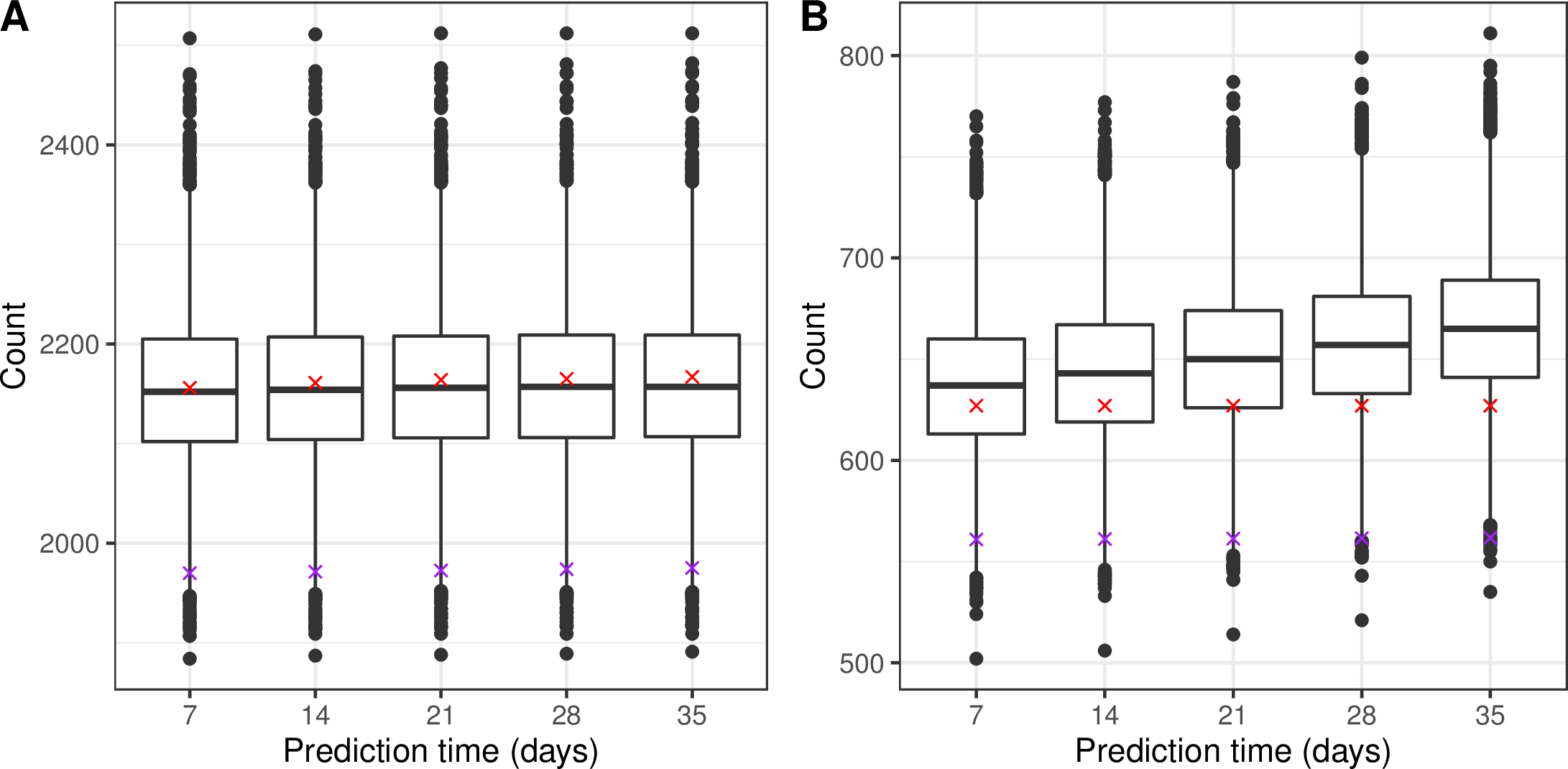

Supplement: S5 Fig — S5A Fig shows cumulative cases of malaria for China and S5B Fig for Eswatini respectively. The red crosses show real number of cumulative cases, the purple crosses show the predictions from the growth model and the box and whisker plot show predictions from the 10,000 simulations. The box shows the interquartile range and the whiskers show 1.5 times the interquartile range above and below the 25th and 75th percentile. (TIF) [file pcbi.1008830.s009.tif]
