# Supplementary material for: Using Hawkes Processes to model imported and local malaria cases in near-elimination settings
Source: PLoS Comput Biol. 2021 Apr 1;17(4):e1008830. doi: 10.1371/journal.pcbi.1008830 (PMC8043404; doi:10.1371/journal.pcbi.1008830)
Supplement: S1 Table — Uncertainty was calculated using the bootstrap method in Reinhart [34] and Sarma et al. [35]. (PDF) [file pcbi.1008830.s010.pdf]

	Fitted value [95% confidence interval]
α	0.0308 [0.0126, 0.0676]
δ	0.0789 [0.0248, 0.1681]
A	2.1369 [-7.1932, 2.8548]
B	-0.0018 [-0.1689, -0.0014]
M	-0.5836 [-0.8269, 10.2468]
N	0.3262 [-1.0208, 9.8495]
