# Supplementary material for: Using Hawkes Processes to model imported and local malaria cases in near-elimination settings
Source: PLoS Comput Biol. 2021 Apr 1;17(4):e1008830. doi: 10.1371/journal.pcbi.1008830 (PMC8043404; doi:10.1371/journal.pcbi.1008830)
Supplement: S2 Table — Uncertainty was calculated using the bootstrap method in Reinhart [34] and Sarma et al. [35]. (PDF) [file pcbi.1008830.s011.pdf]

	Fitted value [95% confidence interval]
alpha	0.0168 [0.0031, 0.1299]
delta	0.0569 [0.0095, 2.1562]
A	0.4001 [-0.4440, 0.5806]
B	0.0001 [-0.0401, 0.0003]
M	0.3045 [0.2306, 1.5897]
N	-0.1235 [-0.2143, 0.7111]
