# Supplementary material for: Using Hawkes Processes to model imported and local malaria cases in near-elimination settings
Source: PLoS Comput Biol. 2021 Apr 1;17(4):e1008830. doi: 10.1371/journal.pcbi.1008830 (PMC8043404; doi:10.1371/journal.pcbi.1008830)
Supplement: Attachment — Submitted filename: Comments.pdf [file pcbi.1008830.s012.pdf]

OVERVIEW

This manuscript adapts an existing mathematical approach, previously used in other disciplines, to model the transmission of malaria in near-elimination settings. The authors provide a rigorous description of the model and then fit it to two separate malaria data sets – *Plasmodium falciparum* in Eswatini and *Plasmodium vivax* in China. They compare their fits to the cumulative incidence in both settings, demonstrating good agreement between the fitted model and the data. Finally, using their fitted model, the authors compare short-term forecasts from their fitted model to additional data from Eswatini and China.

The manuscript offers a novel approach to measure transmission of malaria in near-elimination settings. This approach is distinct from traditionally used network-based approaches to estimate R_c and has a clear advantage in being able to generate short-term forecasts. While network-based approaches offer a retrospective snapshot of transmission, the authors' Hawkes-based approach allows for forward simulation, enabling modelers and policy-makers to generate short-term forecasts of malaria incidence.

In its present form, the manuscript has some limitations that should first be addressed. With the development of any novel inference algorithm, it is common practice to demonstrate proof of concept on simulated data. This appears particularly true in this instance as the authors note that the optimization surface is complex and apt to get stuck in local minima. Second, the authors do not consider unobserved infections and do not consider how robust their estimates are to this. Finally, the estimates of the serial interval for both *P. falciparum* and *P. vivax* are considerably shorter than previous estimates in the literature.

COMMENTS

Introduction

[Lines 18-25] In distinguishing their method from the approach of Routledge *et al.*, the authors contend that the approach of Routledge *et al*. requires very good data sets to generate model predictions. However, both this study and the studies from Routledge *et al.* make use of line list data. It is unclear to me how the data vary between the two studies and if this is an advantage of the current study.

[Lines 52-59] In low transmission settings, estimates of EIR and parasite prevalence are unreliable metrics of transmission. The authors proposed, as a result, that Hawkes Process models will be particularly useful in these settings. It is not clear to me the link between these two arguments. Is this because Hawkes Process models make use of incidence data instead?

[Line 64] Swaziland is now known as Eswatini. Please correct here and elsewhere.

Background

[Line 93] The authors use the notation λ_N to denote the intensity function of HawkesN. In eq. (2), however, the notation is λ^H. Please be consistent.

[Lines 94-105] When using variables to introduce the HawkesN Process and its interpretation, please make sure to define them. It is unclear from the text alone what mu, beta, alpha, delta, and theta signify.

[Line 103] How does a "vanilla" Hawkes Process differ from the univariate Hawkes Process introduced in eq. (1)?

Methods

[Eq. (7)] Is $\alpha(t - t_i)$ the functional evaluation of α with respect to $t - t_i$ or is it the product of two terms α and $(t - t_i)$? It is not clear from the equation, as the functional evaluation notation is used on the left-hand side of the equation. The same question applies to $\delta(t - t_i)$.

[Eq. (7)] Why do the authors not allow for seasonality in the force of infection as they do for the time-varying importation? Malaria transmission is seasonal, due to fluctuations in mosquito density, for instance.

[Lines 183-185] The authors note that the observed line list could be recreated from a wide variety of parameter combinations. This underscores the importance of validating your inference algorithm on simulated data. Algorithm 1 can be used to simulated data to which your inference algorithm can be applied. I believe that it would strengthen the overall results to validate your approach on simulated data.

[Eq. (10)] Should R be R_c (as mentioned on Line 190)?

[Line 199 & Eq. (10)] It is unclear to me whether $t_{\text{max intensity}}$ is the timing of the maximum intensity or the magnitude of the maximum intensity. The use of *t* would suggest a time, but the Line 199 indicates otherwise.

[Lines 224-225] Why look at cumulative incidence instead of daily incidence?

[Line 226] Aren't imported malaria cases by definition exogenous, not endogenous?

Results

[Lines 238-239] I have concerns about the estimate of the serial interval for Eswatini and China. It appears that, on average, the authors estimated that the time scale of transmission is approximately 15 days. This seems way shorter than previous estimates in the literature. Churcher *et al*. (2014) and Huber *et al*. (2016) estimate a mean serial interval of approximately 102 days for untreated *P. falciparum* infections and mean serial intervals of 48 and 33 days, respectively, for treated *P. falciparum* infections. In fact, given an extrinsic incubation period, for instance, of 10 days, we would expect a serial interval to be at minimum 22 days for the author's model. This minimum would ignore any delay from mosquito infectiousness to the infectious bite as well as any delay from the infection to the onset of symptoms in the secondary

individual. The serial interval can be shortened by differences in the detection of the primary and secondary individual, but it does not appear to be modeled here.

[Lines 238-239] How do the authors account for the possibility of a relapsing infection in their *P. vivax* data set in China?

[Lines 246-247] Is the seasonal nature of importation estimated for both China and Eswatini consistent with programmatic observations?

[Lines 252-253] How robust would these results be to unobserved infections? Would the authors expect to over- or under-estimate R_c in the presence of unobserved infections? The authors should consider exploring this in a simulation study.

[Lines 252-253] Are the cumulative imported cases those that are classified as imported cases by the National Malaria Control Program in each country? The authors may consider noting that these cases classifications are not necessarily perfectly accurate in the absence of genotyping.

Discussion

[Lines 298-300] Could the inability to reconstruct the initial time series from Eswatini be attributed to the fact that initially there are no imported infections in the time-series and, as a result, there is a delay before local transmission is able to explain the observed incidence? The authors could consider estimating an initial number of imported infections in their model.

[Lines 304-317] Could the authors comment on whether the Hawkes Process model could be extended to incorporate spatial data?
