# Supplementary material for: Using Hawkes Processes to model imported and local malaria cases in near-elimination settings
Source: PLoS Comput Biol. 2021 Apr 1;17(4):e1008830. doi: 10.1371/journal.pcbi.1008830 (PMC8043404; doi:10.1371/journal.pcbi.1008830)
Supplement: Attachment — Submitted filename: Ref-PLOSCompBiol.pdf [file pcbi.1008830.s013.pdf]

Report on : Using Hawkes Processes to model imported and local malaria cases in near elimination settings

by T. Juliette T. Unwin et al.

This paper presents the use of Hawkes processes to model a malaria epidemic. It is limited to a "near–elimination setting", in the sense that the proportion of susceptible individuals in the population is close to 1 (large population, small incidence). In fact the same model could be used in cases of the start of an outbreak. By the way, I am a bit confused since I thought that in the case of malaria, individuals who have been infected in the past have at least a partial immunity. The authors should explain in which cases it is feasible to replace as they do it the "Hawkes$N$ Process" by a "vanilla Hawkes Process".

Since the title contains the words "near elimination setting", I thought that the author would discuss prediction of extinction time. This is not really the case, but could probably be sutied using the model of this paper.

Rather, the paper is a bit less ambitious, and describes a nice model of malaria epidemic, and the authors compare simulations using their model with parameters fitted to the data, and two sets of data.

The paper details the model (in particular the choice of a parametric family of kernels, plus delays), estimation of the maximum of the rate (needed for the simulations), the simulation algorithm, and the procedure of estimation of the parameters.

The paper is vey well written. I particularly liked section 1 of the supplementary material, which is very clear.

As I said, the model looks like a really good model (the same ideas could be used for other epidemic diseases, including the SARS-CoV-2). The comparisons between the simulations using the model and the data are very convincing. Therefore I recommend acceptance of this paper.

I found only two minor errors to be corrected.

1. On page 5 of the paper, in formula (8), the factor $(t - (t_i + \Delta))$ should be replaced by its positive part : $(t - (t_i + \Delta))^+$.

2. On page 1 of the supplementary material, in formula (5), the lower bound of the integral should not be T_n but t_n.
