# Supplementary material for: Using Hawkes Processes to model imported and local malaria cases in near-elimination settings
Source: PLoS Comput Biol. 2021 Apr 1;17(4):e1008830. doi: 10.1371/journal.pcbi.1008830 (PMC8043404; doi:10.1371/journal.pcbi.1008830)
Supplement: Attachment — Submitted filename: pcompbiol-1.pdf [file pcbi.1008830.s014.pdf]

Review of "Using Hawkes Processes to model imported and local malaria cases in near-elimination settings"

September 15, 2020

This paper presents a potentially useful approach for modeling near-extinction diseases using Hawkes processes. I think Hawkes processes are potentially a good fit for this problem, but I'd like to see the results explored in more depth with more thorough checks of the model fit, performance, and diagnostics. See below for further comments.

Main Comments

1. The paper goes to some length to make the simulation algorithm, which requires knowing the upper bound of the intensity function, work. Is there a reason one can't use an algorithm based directly on the cluster structure of the Hawkes process? This would merely require being able to draw from a Rayleigh distribution and being able to integrate it. A suitable simulation algorithm is reviewed as Algorithm 5 of Reinhart (2018).

   Perhaps I'm missing a reason this can't work, in which case that reason should be stated somewhere. If it *can* work, it would obviate the need for the complicated simulation algorithm.

2. The Results section primarily uses graphs and simulations to validate the model fit. But there are plenty of good goodness-of-fit and diagnostic methods for Hawkes processes. For example, one can plot the event times $\{t_i\}$ against the integral

   $$\int_0^{t_i} \lambda(t)\,\mathrm{d}t.$$

   Since that integral is the expected number of events over $[0, t_i)$, the plot should be a diagonal line; deviation from the line suggests a lack of fit. One can also use the time-rescaling theorem (Brown, Barbieri, Ventura, Kass, & Frank, 2002) and test whether the data, when rescaled using the fitted intensity function, is a homogeneous Poisson process.

   There are also proper scoring rules for point processes (Daley & Vere-Jones, 2004), which can help with the comparisons between models.

   I'd appreciate the Results section being expanded to more fully explore the model fit and show relevant diagnostics and metrics so we can assess if it truly does fit.

3. It would also be helpful for the Results section to clearly compare against baseline models, so we can see how much the Hawkes process (incorporating self-excitation and the new features introduced in this paper) improves upon simple methods. It's hard to interpret the results and figures without any point of comparison.

4. I'd appreciate some discussion of how a model such as this could be *used.* A purely temporal model such as this does not tell you, say, where to direct vector control efforts. What motivation underlies this model, besides that modeling is inherently useful?

Minor Issues

1. On page 3, lines 88–90, the authors refer to two examples of Hawkes processes being used in epidemiology. Another example is Meyer, Elias, and Höhle (2012), although, the authors are nonetheless correct that this is not a commonplace tool in the field.

2. On page 3, line 94, presumably N_t is the number of infected individuals *at time t*. And in the equation, the intensity function is presumably the intensity at time t.

3. Equation (5) seems to be missing a "d", as in

$$\log L(\theta) = \sum_{i=1}^{n} \log \lambda(t_i) - \int_0^T \lambda(\tau)\, \mathrm{d}\tau.$$

4. On page 6, lines 179–181, it's specified that analytic directional derivatives of the log-likelihood are given. It's not explicitly stated, but I assume this is because these derivatives were provided to `optim` to speed the optimization?

5. I don't see the number of cases in the China and Swaziland datasets mentioned anywhere. How much data is involved here?

6. Pages 5 and 6 should make clear which parameters (of equations 7–9) are being fit and which are fixed to "known" values.

7. Page 6 mentions that "The likelihood loss function is also non convex [36]." However, reference 36 does not use the word "convex" as far as I can tell, and works with a different form. Have you experienced multiple modes in the optimization specifically? Is this is the right reference?

References

Brown, E., Barbieri, R., Ventura, V., Kass, R. E., & Frank, L. M. (2002). The time-rescaling theorem and its application to neural spike train data analysis. *Neural Computation*, *14*(2), 325–346. doi:10.1162/08997660252741149

Daley, D. J., & Vere-Jones, D. (2004). Scoring probability forecasts for point processes: The entropy score and information gain. *Journal of Applied Probability*, *41*, 297–312. doi:10.1239/jap/1082552206

Meyer, S., Elias, J., & Höhle, M. (2012). A space–time conditional intensity model for invasive meningococcal disease occurrence. *Biometrics*, *68*(2), 607–616. doi:10.1111/j.1541-0420.2011.01684.x

Reinhart, A. (2018). A review of self-exciting spatio-temporal point processes and their applications. *Statistical Science*, *33*(3), 299–318. doi:10.1214/17-STS629
