# Supplementary material for: Using Hawkes Processes to model imported and local malaria cases in near-elimination settings
Source: PLoS Comput Biol. 2021 Apr 1;17(4):e1008830. doi: 10.1371/journal.pcbi.1008830 (PMC8043404; doi:10.1371/journal.pcbi.1008830)
Supplement: Attachment — Submitted filename: reviewers comments.pdf [file pcbi.1008830.s015.pdf]

Reviewers Comments - "Using Hawkes Processes to model imported and local malaria cases in near-elimination settings"

Reviewer comments are in black and our responses are indicated in blue.

Reviewer 1:

This paper presents a potentially useful approach for modeling near-extinction diseases using Hawkes processes. I think Hawkes processes are potentially a good fit for this problem, but I'd like to see the results explored in more depth with more thorough checks of the model fit, performance, and diagnostics. See below for further comments.

We would like to thank reviewer one for their comments on this manuscript. Following the comments below, which we address in turn, we have expanded our results section and performed goodness of fit tests for our models.

Main Comments

1) The paper goes to some length to make the simulation algorithm, which requires knowing the upper bound of the intensity function, work. Is there a reason one can't use an algorithm based directly on the cluster structure of the Hawkes process? This would merely require being able to draw from a Rayleigh distribution and being able to integrate it. A suitable simulation algorithm is reviewed as Algorithm 5 of Reinhart (2018). Perhaps I'm missing a reason this can't work, in which case that reason should be stated somewhere. If it can work, it would obviate the need for the complicated simulation algorithm.

Thank you for your observation. The algorithms pointed out by this reviewer is a valid alternative to our work. In short, algorithm 5 from Reinhart (2018) proposes a three-step approach:

- First, simulate immigrants from the exogenous intensity (using a non-homogeneous Poisson process);
- For each event in a queue, sample the number of direct offspring from Poisson distribution of param integral of the kernel;
- Sample event times from the kernel normalized to be a pdf -- using the Inverse transform sampling.

We have chosen to develop our current approach based on rejection sampling for the following reasons.

- The inverse transform sampling is efficient when the inverse of the CDF has a closed-form solution, and when the kernel is fixed (does not depend on parent- and external-factors). However, when the kernel does not have a closed-form solution for the inverse, or when external factors occur which modulate the shape of the kernel (say authorities apply control of the spread, extermination of mosquitoes) the inverse requires numerical computation for each event, which severely reduces the effectiveness of the

approach. Furthermore, it is unclear whether the approach can be applied when the shape of the kernel changes as it unfolds (as opposed to being fixed from the occurrence of the parent and until t = infinity). This latter point is particularly important for future work in which interventions are applied during the time an individual is infectious (for example isolating the individual).

- Because in the end, all event times > T will be removed, algorithm 5 from Reinhart (2018) is more useful when sampling until a large time T. For small T, there might be many events requiring being removed.

A rejection sampling approach alleviates the above two shortcomings at the cost of multiple sampling and rejections. This cost is greatly reduced when an adequate maximum can be determined as in our proposed approach.

2) The Results section primarily uses graphs and simulations to validate the model fit. But there are plenty of good goodness-of-fit and diagnostic methods for Hawkes processes. For example, one can plot the event times $\{ti\}$ against the integral $\int\lambda(t)\mathrm{d}t$. Since that integral is the expected number of events over [0, ti), the plot should be a diagonal line; deviation from the line suggests a lack of fit. One can also use the time- rescaling theorem (Brown, Barbieri, Ventura, Kass, & Frank, 2002) and test whether the data, when rescaled using the fitted intensity function, is a homogeneous Poisson process. There are also proper scoring rules for point processes (Daley & Vere-Jones, 2004), which can help with the comparisons between models. I'd appreciate the Results section being expanded to more fully explore the model fit and show relevant diagnostics and metrics so we can assess if it truly does fit.

Thanks for your suggestion about goodness of fit tests. We agree with you that including these has strengthened our results section. Our background intensity (\mu) is time varying so we do not believe a plot of event times against the integral of our intensity should be a diagonal line. We have attached the results of our event times plotted against the integral of our intensity below in Figure 1. It is obvious from these plots that the integral of the intensity follows the sinusoidal nature of our \mu.

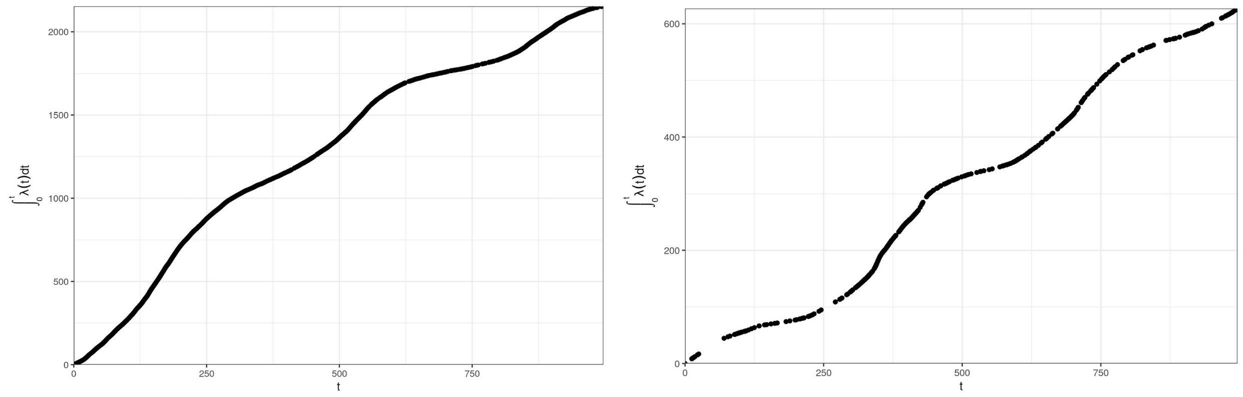

Figure 1: The integral of the intensity at event times for the China dataset (left) and Swaziland/Eswatini dataset (right).

We have included Kolmogorov-Smirnov (KS) tests and quantile--quantile (Q--Q) plots using the time-rescaling method in Brown et al. (2020) in Figures 2, 4 and Supplementary Figure 1. These plots show that our fit for the China data is good as our data lies between the confidence intervals but is less good for Eswatini. Please note we now use the correct name for “Swaziland” in our manuscript. This is inline with our discussion section and the other results in Figures 5 and S2 where our model misses the sharp increase in importations between days 350 and 400 approximately. We also expect more reporting errors in the Eswatini data set, which would reflect in the worse fit.

3. It would also be helpful for the Results section to clearly compare against baseline models, so we can see how much the Hawkes process (incorporating self-excitation and the new features introduced in this paper) improves upon simple methods. It’s hard to interpret the results and figures without any point of comparison.

We agree that comparing our results with baseline models are important in interpreting results. We have compared our model fits to a simple parametric growth model from the *growthrates* R package and an exponential kernel without a delay. Comparisons of these models are now included in Figures 5, 6 and S2 and S3. We find that our model performs better than the parametric growth model in terms of fit and predictive power. It is possible to split out importations using the exponential kernel but not from the parametric growth model, which is a benefit of our methodology. Countries usually rely on patients declaring their travel history to work out if the case is an importation or not.

4. I’d appreciate some discussion of how a model such as this could be used. A purely temporal model such as this does not tell you, say, where to direct vector control efforts. What motivation underlies this model, besides that modeling is inherently useful?

As mentioned above, it is very difficult to work out what proportion of cases are acquired within a country at the national level. Ministries rely on patients self-reporting travel history, which may not be disclosed accurately, may be forgotten to be asked and when accurate may not result in an accurate determination of where the infection truly occurred (Huber (2020)). This has become particularly pertinent during the COVID-19 pandemic where travel restrictions have been in place and people do not wish to declare their movements. Knowing what proportion of cases are coming from importations is important so that the relative effort can be put into blocking local transmission (i.e., through vector control) versus trying to reduce importation in travelers or just offering case management services to those who may have returned with infection In addition, a second application is to simply track transmission rates accurately at the national level, this is hard to do when you know that most of the observed malaria may not be locally acquired. This is important for knowing whether interventions are working and whether the situation is improving or deteriorating. We have added this sentiment to the conclusions.

The next stage of this research is to develop a spatial model for more fine grained control, but traditional methods for spatial-temporal Hawkes have not generated satisfactory results so far.

Minor Issues

1. On page 3, lines 88–90, the authors refer to two examples of Hawkes processes being used in epidemiology. Another example is Meyer, Elias, and Höhle (2012), although, the authors are nonetheless correct that this is not a commonplace tool in the field.

Thanks for this additional reference - we have added it in.

2. On page 3, line 94, presumably N_t is the number of infected individuals at time t. And in the equation, the intensity function is presumably the intensity at time t

Sorry for this unclear notification. We have now updated it to reflect that lambda is evaluated at time t and that Nt refers to the number of infections that occurred before or at time t. This assumes immunity exists for the disease in question.

3. Equation (5) seems to be missing a “d”, as in $nT \log L(\theta) = \sum \log \lambda(t_i) - \int \lambda(\tau) \mathrm{d}\tau$.

Thanks for noticing this result of a missing latex package. This has been corrected although we have now moved some of the background into the supplementary information to not distract from the main message of the paper.

4. On page 6, lines 179–181, it’s specified that analytic directional derivatives of the log-likelihood are given. It’s not explicitly stated, but I assume this is because these derivatives were provided to optim to speed the optimization?

Yes - the analytic directional derivatives were not needed, but help with the speed of the optimiser if included. This has been added to the text in the “Fitting Hawkes Processes” subsection.

5. I don’t see the number of cases in the China and Swaziland datasets mentioned anywhere. How much data is involved here?

Sorry for omitting these originally. We have included the numbers of cases in each data set at the start of the results section. There are 2153 cases in the China dataset and 627 cases in the Swaziland/Eswatini dataset.

6. Pages 5 and 6 should make clear which parameters (of equations 7–9) are being fit and which are fixed to “known” values.

Sorry for this lack in clarity. We have expanded the initial explanation on line 162 of the old manuscript draft to explain which values we are fitting to in each equation. We also repeat this

at the beginning of the Fitting Hawkes Processes section. We fit to all parameters (\alpha, \delta, A, B, M and N) except \Delta, which we fix to 15 days.

7. Page 6 mentions that “The likelihood loss function is also non convex [36].” How- ever, reference 36 does not use the word “convex” as far as I can tell, and works with a different form. Have you experienced multiple modes in the optimization specifically? Is this is the right reference?

Thank you for bringing this to our attention. The reference was remembered incorrectly and is for a power law kernel, where authors have previous experience. Menon and Lee (2018) and Lime and Choi (2018) suggest that Hawkes models are not necessarily convex and have been added to the manuscript instead. We have further investigated the convexity of our negative log-likelihood and found that the eigenvalues of our hessian (returned numerically by the optim solver) do not all have the same sign and so our optimisation finds a saddle point, thus the objective function is not convex.

Reviewer 2:

This paper presents the use of Hawkes processes to model a malaria epidemic. It is limited to a “near–elimination setting”, in the sense that the proportion of susceptible individuals in the population is close to 1 (large population, small incidence). In fact the same model could be used in cases of the start of an outbreak.

Yes - we believe this method is very versatile and the semi-mechanistic part of the model can be adapted to best incorporate disease specific information. We see added value from how we can incorporate malaria from importation (or other diseases from importations or zoonotic spill over), which is not of any significance in a high transmission environment.

By the way, I am a bit confused since I thought that in the case of malaria, individuals who have been infected in the past have at least a partial immunity. The authors should explain in which cases it is feasible to replace as they do it the “HawkesN Process” by a “vanilla Hawkes Process”.

Thanks for this comment and sorry for our lack of clarity. You are correct that individuals who are infected in the past have at least partial immunity to malaria. However, because we are in a near elimination setting where the number of people who have been infected (and so have partial immunity) is much smaller than the total susceptible population, the (1- N_t/N) is approximately equal to 1 and we can approximate the “HawkesN Process” by the “vanilla” Hawkes Process regime. We have updated the description in the text.

Since the title contains the words “near elimination setting”, I thought that the author would discuss prediction of extinction time. This is not really the case, but could probably be suited using the model of this paper.

In this paper, we are measuring transmission rates in environments that have meaningful rates of importation, e.g in our case studies of China and Eswatini. That means "extinction" overall does not happen because there is always malaria observed, since importation happens continually and some level of transmission occurs from those imported infections. Each mini-outbreak will extinguish, but malaria persists overall. The benefit of this method is that it lets us measure transmission in such an environment, when we can't be sure which cases are truly imported and which are locally acquired, and thus have a means of evaluating the success of our local control measures.

Rather, the paper is a bit less ambitious, and describes a nice model of malaria epidemic, and the authors compare simulations using their model with parameters fitted to the data, and two sets of data. The paper details the model (in particular the choice of a parametric family of kernels, plus delays), estimation of the maximum of the rate (needed for the simula- tions), the simulation algorithm, and the procedure of estimation of the parameters. The paper is very well written. I particularly liked section 1 of the supplementary material, which is very clear.

Thank you for providing a good summary of the paper and appreciating the effort we put into ensuring clarity in our narrative. We do not consider this as a single malaria epidemic but a model of a "sink" area which is continually replenished by importation.

As I said, the model looks like a really good model (the same ideas could be used for other epidemic diseases, including the SARS-CoV-2). The comparisons between the simulations using the model and the data are very convincing. Therefore I recommend acceptance of this paper.

I found only two minor errors to be corrected.

1. On page 5 of the paper, in formula (8), the factor (t − (ti + ∆)) should be + replaced by its positive part : (t − (ti + ∆))^{+}

Thanks - we have instead opted for the notation \forall t>t_{i} + \Delta and updated the new equations 4 and 5.

2. On page 1 of the supplementary material, in formula (5), the lower bound of the integral should not be Tn but tn.

Thanks for spotting this - we have corrected it.

Reviewer 3:

This manuscript adapts an existing mathematical approach, previously used in other disciplines, to model the transmission of malaria in near-elimination settings. The authors provide a rigorous description of the model and then fit it to two separate malaria data sets – *Plasmodium falciparum* in Eswatini and *Plasmodium vivax* in China. They compare their fits to the cumulative incidence in both settings, demonstrating good agreement between the fitted model and the data. Finally, using their fitted model, the authors compare short-term forecasts from their fitted model to additional data from Eswatini and China.

Thank you for your fair summary of our manuscript.

The manuscript offers a novel approach to measure transmission of malaria in near-elimination settings. This approach is distinct from traditionally used network-based approaches to estimate R_c and has a clear advantage in being able to generate short-term forecasts. While network-based approaches offer a retrospective snapshot of transmission, the authors' Hawkes-based approach allows for forward simulation, enabling modelers and policy-makers to generate short-term forecasts of malaria incidence.

We too believe that Hawkes Processes provide a new opportunity for short-term forecasts.

In its present form, the manuscript has some limitations that should first be addressed. With the development of any novel inference algorithm, it is common practice to demonstrate proof of concept on simulated data. This appears particularly true in this instance as the authors note that the optimization surface is complex and apt to get stuck in local minima.

Thank you for noticing this omission. We have now included a simulated data section in our results. We now assume some parameters (the best fit parameters from our Eswatini data set) and simulate 10,000 simulations from them. We then re-fit each of these simulations to show the fitting ability of our method and provide goodness of fit tests for a subset of these fits.

Second, the authors do not consider unobserved infections and do not consider how robust their estimates are to this.

We also appreciate the importance of this. We have investigated this in our simulated data section and found that our model is robust up to 10% of data missing for all parameters and further for some parameters. We plan to explore this further in subsequent work.

Finally, the estimates of the serial interval for both *P. falciparum* and *P. vivax* are considerably shorter than previous estimates in the literature.

Thank you for pointing this out. We have thought more about this and we address your concerns where you mention them again in the Results section comments.

COMMENTS Introduction

[Lines 18-25] In distinguishing their method from the approach of Routledge *et al*., the authors contend that the approach of Routledge *et al*. requires very good data sets to generate model predictions. However, both this study and the studies from Routledge *et al.* make use of line list data. It is unclear to me how the data vary between the two studies and if this is an advantage of the current study.

Thanks for raising this concern. We do use the same China linelist data from Routledge et al. but we believe that the Hawkes Process method is more robust to missing data than the network model (in Routledge et al.). We now show in our manuscript that Hawkes Processes are robust to missing data to some extent (see Figure 3) , whereas the network method relies on having a complete line list. This means we are able to apply our methods to the Eswatini data set, which we believe is less complete than the China dataset. We have further addressed this in the discussion section. In addition, by using Hawkes Processes, we are able to predict if cases are importations or not without using labelled cases and considering seasonality and travel history.

[Lines 52-59] In low transmission settings, estimates of EIR and parasite prevalence are unreliable metrics of transmission. The authors proposed, as a result, that Hawkes Process models will be particularly useful in these settings. It is not clear to me the link between these two arguments. Is this because Hawkes Process models make use of incidence data instead?

Yes - sorry for this lack of clarity. We do not require population prevalence estimates in our model, just a line list (incidence data). We have addressed this in the introduction where we say Hawkes Processes are fit to incidence data.

[Line 64] Swaziland is now known as Eswatini. Please correct here and elsewhere.

Thanks for raising this, we are sorry for this omission.

Background

[Line 93] The authors use the notation IN to denote the intensity function of HawkesN. In eq. (2), however, the notation is IH. Please be consistent.

Thanks for identifying this inconsistency in notification. We have rectified it.

[Lines 94-105] When using variables to introduce the HawkesN Process and its interpretation, please make sure to define them. It is unclear from the text alone what mu, beta, alpha, delta, and theta signify.

Sorry for not fully defining our parameters. The parameters in the HawkesN model are now fully defined after it is introduced in equation 2 and the parameters for the SIR model that it is compared to are also defined in the same paragraph.

[Line 103] How does a “vanilla” Hawkes Process differ from the univariate Hawkes Process introduced in eq. (1)?

The “vanilla” Hawkes Process is the same as the univariate Hawkes Process. We have ensured consistency of terminology throughout our manuscript.

Methods

[Eq. (7)] Is a(t – ti) the functional evaluation of a with respect to t – ti or is it the product of two terms a and (t – ti)? It is not clear from the equation, as the functional evaluation notation is used on the left-hand side of the equation. The same question applies to d(t – ti).

Thanks for picking up the mix of notations throughout the manuscript. We use, for example, \mu(t) to denote \mu evaluated at time t, but in these cases we mean a*(t-ti). We have clarified this.

[Eq. (7)] Why do the authors not allow for seasonality in the force of infection as they do for the time-varying importation? Malaria transmission is seasonal, due to fluctuations in mosquito density, for instance.

Thank you for this thought, but we do not consider it necessary in our applications. Since we are in a near elimination setting, the seasonal importation is driving transmission in our datasets opposed to the person-to-person transmission. It could be possible to further expand our model to include marks, which would allow the magnitude of our infections to change between cases, in situations where this is necessary. However, we do not think this is necessary here because there is little noticeable seasonality in the numbers of non-imported cases.

[Lines 183-185] The authors note that the observed line list could be recreated from a wide variety of parameter combinations. This underscores the importance of validating your inference algorithm on simulated data. Algorithm 1 can be used to simulated data to which your inference algorithm can be applied. I believe that it would strengthen the overall results to validate your approach on simulated data.

Thank you for your suggestion about simulated data - as explained above we now show in the results section that the Hawkes model performs well on simulated data. In our first draft we did include simulated data but only to show the recreation of our epicurves and not to show how well our model fits to the data.

[Eq. (10)] Should R be Rc (as mentioned on Line 190)?

Yes - thanks for picking up the missing subscript. We have added it in now.

[Line 199 & Eq. (10)] It is unclear to me whether tmax intensity is the timing of the maximum intensity or the magnitude of the maximum intensity. The use of *t* would suggest a time, but the Line 199 indicates otherwise.

Sorry for this confusion - tmax is the time of the maximum intensity and not the magnitude. We have edited the text accordingly.

[Lines 224-225] Why look at cumulative incidence instead of daily incidence?

We originally presented cumulative incidence because we felt this was the natural output from the Hawkes Process as it is a counting process. We now present daily incidence in our results and cumulative cases in our supplementary information.

[Line 226] Aren't imported malaria cases by definition exogenous, not endogenous?

We have removed the word endogenous from the old line 226. It was originally included to try and distinguish between the imported malaria cases and the within country transmission using the terminology earlier in the paper. However, this is confusing terminology with malaria.

Results

[Lines 238-239] I have concerns about the estimate of the serial interval for Eswatini and China. It appears that, on average, the authors estimated that the time scale of transmission is approximately 15 days. This seems way shorter than previous estimates in the literature. Churcher *et al*. (2014) and Huber *et al*. (2016) estimate a mean serial interval of approximately 102 days for untreated *P. falciparum* infections and mean serial intervals of 48 and 33 days, respectively, for treated *P. falciparum* infections. In fact, given an extrinsic incubation period, for instance, of 10 days, we would expect a serial interval to be at minimum 22 days for the author's model. This minimum would ignore any delay from mosquito infectiousness to the infectious bite as well as any delay from the infection to the onset of symptoms in the secondary individual. The serial interval can be shortened by differences in the detection of the primary and secondary individual, but it does not appear to be modeled here.

Thank you for raising your concern here. We assume everyone in our data set is treated because they have reported to a health care facility to be recorded on the linelist. This has been emphasised in the manuscript. We have also had considerable discussion around the shape and duration of our fitted kernel in light of this comment. First, we believe that we were wrong in calling the intensity from our shifted Rayleigh kernel the serial interval distribution. What we have is actually the intensity between symptom onset and a second person symptom onsetting from an infection from person one. We believe this should have a similar shape to the serial interval (delay followed by a Rayleigh kernel). After re-fitting our kernel including a correction to the analytic gradients the duration of our kernel, the time scales of transmission have increased by approximately 10 days. Assuming an extrinsic incubation period of 10 days, our serial interval now would be approaching 30 days (10 + 20 from our kernel).

We were initially unsure if our original assumption of the delay period (zero intensity in our kernel) was correct so we adapted our code to allow the user to fit the length of delay in the model. We found that our optimisation problem was over-defined when we were fitting to this additional parameter and we got trapped in a large flat minima where similar values of our log-likelihood were returned. This meant we decided to fix the delay to be 15 as in literature (as

in Routledge et al 2020), to reflect the minimum time from a mosquito biting an individual and becoming infectious and a second person becoming infectious, and could consistently approximate our other model parameters including the duration a person remained infectious.

[Lines 238-239] How do the authors account for the possibility of a relapsing infection in their *P. vivax* data set in China?

Thank you for raising this point. We have assumed in our model that each infection is a new infection opposed to a relapsed infection and is a limitation of our approach. We have added into the limitation section of the manuscript. We do not believe this would have a large impact on the model results because we find a very large proportion of the China dataset comes from importations, which is in-line with near-elimination modelling. This is consistent with the labelled data.

[Lines 246-247] Is the seasonal nature of importation estimated for both China and Eswatini consistent with programmatic observations?

We believe the seasonal importations are consistent with programmatic observations - in Figure 5 and S2 we show we recreate the seasonal fluctuations in the importations from the labelled line lists for both China and Eswatini as well as the overall cases. China's rainy season is April through to September, which is consistent with the increase in importations around day 150 (assuming importations from bordering countries with similar climates) and a trough in the intensity towards the end/beginning of the year (Routledge(2020)). Past analysis (e.g. Tejedor-Garavito (2017)) suggests importation in Eswatini happens because residents travel to visit family in higher endemic Mozambique around the December holidays, then return to Eswatini with infections in January - February. Those infections then appear to seed transmission outbreaks over the course of the year.

[Lines 252-253] How robust would these results be to unobserved infections? Would the authors expect to over- or under-estimate *Rc* in the presence of unobserved infections? The authors should consider exploring this in a simulation study.

Thank you for suggesting this. We have now incorporated a section in our results on simulated data and address the issue of under reporting or not observing all infections. We find that our parameter estimates are robust for up to 10% of data missing for all parameters and further for some parameters. We also now provide uncertainty intervals around our Rc calculation.

[Lines 252-253] Are the cumulative imported cases those that are classified as imported cases by the National Malaria Control Program in each country? The authors may consider noting that these cases classifications are not necessarily perfectly accurate in the absence of genotyping.

The cumulative imported cases are those classified by the National malaria control program. We agree that these might not be correct since they can be based on self reporting travel history (Huber (2020)). This highlights one of the main benefits of this method because we offer

an alternative method for disentangling the contribution from importations and within country transmission. Additional emphasis on this has been added into the manuscript.

Discussion

[Lines 298-300] Could the inability to reconstruct the initial time series from Eswatini be attributed to the fact that initially there are no imported infections in the time-series and, as a result, there is a delay before local transmission is able to explain the observed incidence? The authors could consider estimating an initial number of imported infections in their model.

We believe that this is exactly the problem encountered with the Eswatini data set. Because there are very few importations in the first year, our kernel has to compensate with either a larger magnitude or increase the serial interval. This then increases the amount of cases from the kernel later in our time series. We could have seeded our model with multiple cases or fit a different set of parameters to year 1 and years 2 and 3. However, we did neither of these because we didn't have any evidence from the data collection that things had changed over these time periods and equally the problem could have been poor reporting. We wanted to show how good a fit was possible in line with these uncertainties. With our future work we aim to collaborate closely with in-country partners to adjust the semi-mechanistic parts inline with their expertise.

[Lines 304-317] Could the authors comment on whether the Hawkes Process model could be extended to incorporate spatial data?

We believe that the Hawkes Process can be made spatial and it is the next avenue we will be exploring. Unfortunately, we have not found the spatial component is identifiable with the existing methods such as using multivariate Hawkes with an exponentially decaying kernel.

Reviewer 4:

This manuscript applies temporal Hawkes process models to malaria occurrence in China and Swaziland. Such self-exciting point process models are "relatively" new for applications in infectious disease epidemiology, if "relatively" means like 10 years of research or so, and they are not yet applied frequently in this field. A recent review is given by Reinhart (2018, https://doi.org/10.1214/17-STS629), with a focus on spatio-temporal extensions of the simple Hawkes process.

We thank you for your summary.

My main concern with this manuscript is that the purely temporal Hawkes model presented here is somehow obsolete as spatio-temporal versions have already been established and applied, also for infectious diseases. The manuscript seems to completely *ignore* these methods and applications, concentrates on a simple temporal Hawkes model albeit saying that "malaria is a complex disease to model [...] the inoculation rate varies greatly in space". All the more important is a spatially structured model, in particular when investigating the probability of fade-out. It would be interesting to see the suggested temporal Rayleigh kernel with delay

applied in a spatio-temporal model and compare it to exponential and nonparametric triggering functions, respectively.

We agree with you that a spatial temporal Hawkes Process model would be the gold standard model for malaria transmission and is the aim of our subsequent work. We considered using standard methods such as an exponential kernel for the spatial dimension but have found these methods unsuitable for our data and sometimes we do not have exact spatial information but instead an administrative unit. Our method under-development should hopefully be able to incorporate both types of spatial data. We believe that a temporal Hawkes Process model for malaria is a good addition to literature because we are able to use it to make short term predictions and to also split out contributions from importions, which is of key interest to policy makers in countries close to elimination.

The other main issues are potential errors in likelihood maximization and a lack of confidence intervals for the parameter estimates.

Thank you for raising these concerns. We have addressed them in turn below.

Major issues

1. The manuscript suggests that Hawkes process models are "relatively". I'd argue that such models aren't that rare in the literature, certainly if we also look for more advanced spatio-temporal Hawkes models. The following research seems to have been ignored:

a) Methodological/Software-focussed: An implementation for temporal Hawkes processes is provided by the R package "PtProcess" (already ~10 years old). A multivariate temporal Hawkes process for infectious disease transmission across a network of individuals was proposed by Höhle (2009, https://doi.org/10.1002/bimj.200900050) and a spatio-temporal self-exciting process by Meyer et al (2012, https://doi.org/10.1111/j.1541-0420.2011.01684.x) with implementations in the R package "surveillance", whereas Almutiry and Deardon (2019, https://doi.org/10.1515/ijb-2017-0092) focus on individual-level and network effects and assume a time-constant triggering kernel, with implementation in the R package "EpiILMCT".

b) Applications:

https://doi.org/10.1198/jasa.2011.ap09546 (crimes, many more publications in this field)

https://doi.org/10.1080/01621459.2011.641402 (invasive plant species)

https://doi.org/10.1111/j.1541-0420.2011.01684.x (invasive meningococcal disease)

https://doi.org/10.1080/01621459.2015.1135802 (e-mail communication behaviour)

https://doi.org/10.1098/rspb.2016.0952 (spread of a wildlife pathogen)

https://doi.org/10.1080/02664763.2020.1825646 (Ebola)

From my quick search for applications, I would agree that only "a few people now use Hawkes Processes *for epidemiological modelling*", but the modelling approach per se is really no longer in its infancy. Furthermore, there are examples of using a seasonal exogeneous effect (just like this) in the literature (p. 5, l. 160), e.g., in the aforementioned meningococcal disease application.

Thank you for these extra references. We agree that Hawkes Processes have been applied to a wide variety of applications and have added emphasis on this in the background section, where we already reference several of these. Our aim was to show that Hawkes Processes are well founded, but relatively new to epidemiological modeling. We have added a reference to the meningococcal disease application too.

2. The manuscript mentions Kelly et al (2019) for a recent Hawkes process modelling approach. A nice feature of that work is that no particular functional form is assumed for the triggering kernel; instead a step function is estimated and smoothed. The authors should really consider such a nonparametric approach as a means of validating the Rayleigh kernel parametrization.

We believe one of the benefits of the Hawkes Process is that it is semi-mechanistic and we can encode well researched mechanisms into our statistical processes. Literature e.g. Routledge et al (2016, 2018), Huber et al (2016) (https://malariajournal.biomedcentral.com/articles/10.1186/s12936-016-1537-6) uses a serial interval distribution similar to our kernel, which we wished to incorporate in our method.

3. The authors suggest to use a kernel with delay to account for the latent period. The current approach has two problems:

a) the kernel is exactly 0 until day 12, when it experiences a sharp increase. A smooth increase seems to be more realistic, in particular because these 12 days won't hold for every case.

We have taken from literature the delay to be 15 days (Routledge 2020). This is the minimum time between infection and suitable numbers of gametocytes in the blood to lead to symptom onset. Then we allow our kernel to increase as is standard in the field.

b) for the kernel to make sense, wouldn't the dates t_i need to correspond to the day the person got infected? The authors say in the discussion that "they are recorded by the day of presentation of symptoms".

Thanks for this comment. We confirm we are fitting to “date of symptoms onset” - individuals, in the China dataset, were asked for the date of symptoms onset when they reported (Routledge et al (2020)). In line with this, we still believe the delay is necessary due to the role the mosquito

plays in malaria transmission. Despite person A being infectious immediately at symptoms onset, person B cannot become infectious from an infection originating from person A for the 15 days it takes for the mosquito to become infectious, pass on the parasites and for them to infect a human.

4. I was really surprised to read that numerical log-likelihood maximization suffered from convergence problems for this model (the parameter space isn't really "complex", l. 332) and these relatively large data sets. I suspected that the gradient might be wrongly derived or implemented. As it turns out, the analytic gradient implemented for `delta` disagrees with a numerical approximation. Running `vignette("fitting")` from the authors' R package and then

set.seed(1)

par <- c(alpha = runif(1, 0, 1), delta = runif(1, 0, 1), A = runif(1, 0, 1), B = runif(1, 0, 1))

maxLik::compareDerivatives(neg_log_likelihood, ray_derivatives, t0 = par, events = events, delay = delay, kernel = ray_kernel, mu_fn = mu_fn, mu_diff_fn = mu_diff_fn,mu_int_fn = mu_int_fn)

I get t0

alpha delta A B

0.2655087 0.3721239 0.5728534 0.9082078

analytic gradient

[,1] [,2] [,3] [,4]

[1,] 217.0904 217.0904 26.45247 566.8895

numeric gradient

alpha delta A B

[1,] 217.0904 -143.9715 26.45247 566.8895

Thank you for investigating this and introducing me to the package “maxLik”. It is very useful and I will definitely use it to check my code henceforth. I had made a mistake in calling the wrong function in my derivatives - I have rectified this and added a test to my suite which uses the makLik function, see commit (03786c5fd39a38ed9e03e3ada344927fa2e59b0f). We still believe the parameter space is complex because the eigenvalues of our Hessian evaluated at our optimal parameter do not all have the same sign, as with the general case of Hawkes with an exponential kernel (we address this further below in 5).

Note that I could only investigate this further because the code was submitted (published, actually) together with the manuscript! What a nice example for the advantage of open science with open source software. :)

Thank you for looking into the vignette and helping me find my mistake. We agree with you that open source code is the future for open science.

I'm curious if the convergence problems go away when the gradient is validated.

Thanks for your suggestions about the gradient. When we fixed the error convergence did improve but when we fitted to the delay period, as mentioned above, we found our system was over-determined and we were in a wide minima and convergence was bad.

5. Related to the above: The authors state that the likelihood loss function is non-convex, referencing Kong et al (2019). I couldn't find this information in the referenced paper. Please verify. From what I know from Rathbun (1996), the log-likelihood of a self-exciting point process is concave if the CIF is linearly parametrized.

We are sorry for the wrong reference. We now reference Menon and Lee (2018) and Lime and Choi (2018), which say that Hawkes Processes may not be convex. We further investigated this by considering the eigenvalues of our hessian, found by our optimisation (with checked gradients). We found one of our eigenvalues is negative when the others are positive, which is indicative of a saddle point and a non-convex log-likelihood. We also notice this behaviour with an exponential kernel and our linear plus sinusoidal forcing term.

6. Again related: Please re-check the model fit and simulation based on the exponential kernel. The finding that simulations from the exponential kernel didn't recreate the data may well suffer from a similar error.

We have re-fit our exponential kernel and updated our analysis. We see that a fit to the exponential kernel provides similar fits, although the Kolmogorov-Smirnov goodness of fit test in Supplementary Figure 1 suggests that the Rayeligh kernel is marginally better.

7. The authors seem to have been careful not to write about *basic* reproduction numbers but "case reproduction numbers". I think it is really worth noting that reproduction numbers estimated from such a branching process with immigration are "adjusted" for infections occurring independently of previously *observed* infections. Please see the discussion of Delamater et al (2019, https://doi.org/10.3201/eid2501.171901) on the importance of communicating what is meant by R. Furthermore, from the referenced work by Routledge et al. it seems that the case reproduction number is decreasing over the years. Have you considered estimating case-specific effects on the triggering rate (as in seismology and in some of the aforementioned point process approaches in the literature) as to model decreasing magnitudes α (and thus R) over the years?

We agree with you about the importance in communicating what the reproduction number means. We believe our R to be the case reproduction number since the branching factor gives

us the expected number secondary cases from a primary case. We agree that it could be possible to fit multiple different kernels over time or use marks to identify different cases but we didn't want to do this without further information about changes in intervention cases or information about different cases receiving different treatment. We assumed all treatment was the same because we did not have any information in our data sets about where treatment was sought.

8. Given that inference on the reproduction number is of scientific interest, its estimate should really be accompanied by a 95% confidence interval to quantify uncertainty. Different methods have been proposed to estimate the variance-covariance matrix of the MLE in such point process models (see, e.g., the aforementioned review by Reinhart). I think for your model a numerical estimate of the Hessian would provide a reasonable basis for confidence intervals (after the analytical gradient has been corrected and validated).

Thank you for your suggestion on including uncertainty in our estimates. We investigated two methods for doing this 1) using the Hessian as you suggested and 2) using re-fitting of our simulation. Since the eigenvalues of our hessian evaluated at our optimal parameters were not all positive, we could not use this method. Therefore, as updated in our methods, we refit our 10,000 simulations and took our 95% confidence intervals to be the 2.5% and 97.5% quartiles. The uncertainty in Rc is given in the manuscript text and the uncertainty in the parameters are given in Supplementary Tables 1 and 2.

9. Isn't underreporting also an issue for Malaria cases? The endogenous contribution will be underestimated if cases are missing in the line list. Statistical inference requires knowledge about the data-generating process; a sophisticated Hawkes model can be useful, but underreporting can bias the parameter estimates.

Yes, underreporting can be an issue for Malaria cases. We believe that the China data set is very good, however we believe there is data missing from the Eswantini dataset. We have included a study into the robustness of the model to underreporting in the simulated data results section. We found that our method is robust up to 10% of data missing for all parameters and further for some, Figure 3.

Minor issues

- The introductory part (including the background section) is relatively long. It reminds me of a textbook or thesis chapter. I'm sure some parts could be shortened. For example, Ogata's thinning algorithm is well known and doesn't need to be explained in detail, at least not as part of the main text. The crucial part is that it requires a (temporary) upper bound for the conditional intensity.

Thank you for this suggestion. Our original aim had been to make this paper easy to follow for those unfamiliar with spatial temporal processes, however, we agree that some of the background section would be better placed in the supplementary material.

- The introduction says that mechanistic models "may make strong assumptions such as the homogeneity of the population". I don't see that this is avoided by the proposed Hawkes model, especially because it is spatially aggregated.

We note this point and have removed it from the manuscript.

- p. 2, l. 35-36: Kelly et al. had a constant μ background in their model so it is wrong to says they used a "model with just an endogenous term".

Thank you for pointing out this omission - we have edited the text to reflect the inclusion of the constant term.

- Eq. 2: λ^H -> $\lambda^H(t)$

Thanks, we’ve added in the (t).

- SI part 1 wouldn't be necessary (you could also just reference a textbook) but is nice to have.

We agree with you that we could have pointed the reader to a textbook. However, our aim was to make it inclusive for readers without access to textbooks and who may be less familiar with the necessary mathematics to follow minimal derivations.

- SI Eq. 31: ∂M -> ∂N.

Thanks we’ve edited.

- Eq. 5: τ -> $d \tau$

Thanks we were missing the commath latex package.

- p. 5, l. 158: Is it true that the upper bound is "no longer trivial to find" just because a constant delay is introduced? Or is non-monotonicity a problem (as suggested in line 198)? It seems to me that the mode of the Rayleigh kernel could still be used, i.e. assuming the value $\phi(1/\sqrt{\delta})$ for all currently infectious individuals ($t_i < t + \Delta$). I understand that the time-varying exogeneous term complicates the choice of a suitable upper bound.

Thanks for this comment - we have thought about this some more and believe our statement still to be true. We know the maximum time of a single kernel with a delay but, depending how close the previous events happened, we can arise at a scenario where the maximum intensity can occur during the delay period of the new infection due to the overlapping delays of previous infections. We aimed to describe this in Supplementary Information Appendix 1 and have edited this section to increase its clarity.

- p. 6, l. 201: delays are denoted by τ here but by Δ in eq. 8

Thanks for pointing out inconsistencies in notation. This has been changed to Δ.

- p. 6, l. 171: please explain Plasmodium vivax.

Plasmodium vivax is one of the common parasites that cause malaria. I have clarified this in the text.

- The data is first mentioned on page 6, but the reader has to wait for Figure 3 on p. 9 to finally see what data we were actually modelling. I always prefer to see the data or a descriptive summary thereof, before thinking about any modelling strategy. I'd suggest to describe the data together with the goal of the analysis earlier in the manuscript.

Thanks for this suggestion, I have added to the description at the end of the introduction to introduce the data and analysis.

- Maybe I've overlooked it, but please mention the total number of infections of the two datasets in the text. I only found out approx. from Figure 3.

We have now included the total number of infections in our two data sets at the start of the "Fitting Hawkes Processes to malaria data" section.

- p. 8, l. 227: \alpha = \delta = 0 contradicts the parameter definition in Eq. 7.

Thanks, we have adjusted the definitions in Eq 7 to include zero.

- Figure 2: The term "serial interval distribution" is misleading in that we don't see a density; the kernel doesn't integrate to 1. (This one is picky, I know. Just ignore this point if you prefer.)

We thank you for this and note your point. We have changed our notation and no longer refer to the kernel as the serial interval distribution.

Sebastian Meyer
