# Supplementary material for: Using Hawkes Processes to model imported and local malaria cases in near-elimination settings
Source: PLoS Comput Biol. 2021 Apr 1;17(4):e1008830. doi: 10.1371/journal.pcbi.1008830 (PMC8043404; doi:10.1371/journal.pcbi.1008830)
Supplement: Attachment — Submitted filename: Comments_Round2.pdf [file pcbi.1008830.s016.pdf]

OVERVIEW

The authors have addressed the concerns that I raised in my initial review. Before I recommend the manuscript for publication, I would like to raise one point and also request some clarifications from the authors regarding the simulation sweep.

COMMENTS

In the caption for Figure 2, the authors note that they only plot kernels where all the parameters lie within the respective 95% quantiles. Can the authors justify their reasoning for doing so as opposed to plotting all of the 10,000 inferred parameter sets to show the full range of inferences that they can obtain using the proposed method? It seems to me that restricting the plots to only those parameters sets for which all parameters lie within the respective 95% quantiles would misrepresent the extent to which the inferences reproduce the simulated kernels. I may be misunderstanding this though.

I appreciate the inclusion of a simulation study to examine how the parameter estimates change with the extent of reporting of cases. There did not appear to be a mention of how R_c estimates change with the extent of reporting of cases. Because much of the strength of the method is its ability to estimate the reproduction number under control, the authors should mention how this quantity is affected by reporting in their simulation studies.

Moreover, it would be useful if the authors examined the full range of underreporting from 10-100% to get a sense of at what point inferences break down and therefore where the use of the method may be most appropriate. Even among near-elimination settings, the quality of surveillance systems varies, so the 70% lower bound of reporting may be overly optimistic in some settings.

[Lines 372-373] The authors should tone down the claim that being able to forecast case counts five weeks in advance would enable policy makers to take action to reduce transmission. While I agree that forecasts are useful, the method, as presented in this manuscript, lacks a spatial comment. As one of the other reviewers noted, without spatial information to guide these forecasts, it would be challenging to take actionable steps to reduce transmission in light of these forecasts.
