# Supplementary material for: Using Hawkes Processes to model imported and local malaria cases in near-elimination settings
Source: PLoS Comput Biol. 2021 Apr 1;17(4):e1008830. doi: 10.1371/journal.pcbi.1008830 (PMC8043404; doi:10.1371/journal.pcbi.1008830)
Supplement: Attachment — Submitted filename: pcompbiol-1-revision.pdf [file pcbi.1008830.s017.pdf]

Review of revision 1 of "Using Hawkes Processes to model imported and local malaria cases in near-elimination settings"

January 5, 2021

I thank the authors for their revisions, which have addressed most of my comments from the first version. As described below, I still have some concerns about the model, primarily about the optimization problems the authors discuss in their reply and about the simulation studies. I think these two problems are connected—better simulation studies may reveal what is going on with the optimization—and the authors should carefully investigate and resolve these issues to ensure confidence in their results.

Main Comments

1. The authors note in their reply that

   > We have further investigated the convexity of our negative log-likelihood and found that the eigenvalues of our hessian (returned numerically by the optim solver) do not all have the same sign and so our optimisation finds a saddle point, thus the objective function is not convex.

   This makes me suspicious. Does this consistently occur regardless of the starting values used in the optimization? And are these eigenvalues with the wrong sign on the same scale as the other eigenvalues, or are they, for instance, 10^{-16}?

   I'm suspicious because (like Reviewer 4) I would be surprised of convergence problems for these models. I'd be extra-surprised if multiple optimization runs from different starting parameter values consistently end up in the same saddle point. I wonder if there is an error in the calculation of the Hessian or of the likelihood, or if the eigenvalues with the wrong sign are very close to zero and actually a result of numerical issues in estimating the Hessian. (In that case, they may indicate non-identifiability in the model, which would be a separate problem to solve.)

   In either case, I think this warrants some double-checking (with different starting values and optimizer settings, and of the gradient and likelihood code). A good strategy to diagnose the problem (though perhaps not necessary to present in the paper, depending on what you find) is to make contour plots of the log-likelihood

near the solution, varying the parameter with the suspicious eigenvalue and another parameter.

If the saddle point issues are real, they should be mentioned in the text as a warning for the interpretation of the results.

2. I appreciate that the authors added simulations to validate that their model can recover the true parameters. The text (pages 6–7) states that

 > We simulate 10,000 sets of events... We then use optim to minimise our log-likelihood and find the optimal values of our simulation from one initial set of parameters for each simulation. We compare these fitted parameters to the initial parameters used for the simulation and run KS tests for a sub-sample of our re-fits to check our goodness of fits.

 I don't understand the procedure being described here. I assume "find the optimal values of our simulation from one initial set of parameters for each simulation" simply means the model is fit to the simulated data. But what are the KS tests testing? The hint is on page 5: "If the model is correct, then, according to the theorem, the difference in intensity between two subsequent events are independent exponential random variables with mean 1."

 This seems to suggest that

$$\lambda(t_{i+1}) - \lambda(t_i) \sim \mathrm{Exp}(1),$$

 which is false. (The difference can easily be negative, for instance.) So I hope the text isn't accurate, and the authors are instead applying the time-rescaling theorem correctly.

3. Also on the topic of the simulation studies, I think what I was really looking for was that the *parameters* are recovered accurately. If different parameter values can result in similar intensity functions, then interpretation of parameters of the model—like the reproduction number—depends on whether the MLE is estimating the parameters well. And if the MLE is finding a saddle point, as the authors note, that seems questionable. (If, on the other hand, the MLE estimates the parameters quite accurately, that suggests it's finding the true maximum, not a saddle point, or that the saddle point is somehow always near the maximum. So I think these points are linked: simulations can shed light on what's going on with the likelihood, and make the implications of the optimization problem clearer.)

 The diagnostics I'm thinking of would include, say, a histogram of estimated values of a parameter from the 10,000 simulations, with a line marking the true value. Or a table showing each true parameter value, plus the mean, median, and quantiles of estimates from the simulation. Either would indicate if the estimation algorithm is indeed working. Reinhart and Greenhouse (2018, Section 4) provide some simulation studies of a related model that illustrate how simulations can be used to illustrate the model's properties.

4. In my previous review, I suggested plotting the event times $\{t_i\}$ against the integral $\int_0^{t_i} \lambda(t)\,\mathrm{d}t$. As the authors correctly note in their reply, this need not be a diagonal line like I implied. What I should have said is that plotting the event *indices* $\{i\}$ against the integral $\int_0^{t_i} \lambda(t)\,\mathrm{d}t$ should yield a diagonal line. This, I think, would be a more useful diagnostic than some of the plots included in Figure 2, which don't really demonstrate whether the model is adequately fitting the observed data.

5. The authors have moved discussion of the simulation algorithm and λ^* to the supplementary information. I think this is a good choice, but I suspect the paragraph at the bottom of page 4 should be adjusted, because it now mentions "the thinning Algorithm" and "λ^* is no longer trivial to find" before the algorithm is introduced. λ^* also no longer seems to be defined in the text.

   (The same paragraph should also specify the units, days, when stating that $\Delta = 15$.)

References

Reinhart, A., & Greenhouse, J. B. (2018). Self-exciting point processes with spatial covariates: Modelling the dynamics of crime. *Journal of the Royal Statistical Society: Series C (Applied Statistics)*, *67*(5), 1305–1329. doi:10.1111/rssc.12277
