# Supplementary material for: Using Hawkes Processes to model imported and local malaria cases in near-elimination settings
Source: PLoS Comput Biol. 2021 Apr 1;17(4):e1008830. doi: 10.1371/journal.pcbi.1008830 (PMC8043404; doi:10.1371/journal.pcbi.1008830)
Supplement: Attachment — Submitted filename: Hawkes reviewers comments 2.pdf [file pcbi.1008830.s018.pdf]

Reviewer comments "Using Hawkes Processes to model imported and local malaria cases in near-elimination settings"

We would like to thank all four reviewers for their comments about our manuscript, which have significantly improved our submission. Reviewers comments are shown in black and our replies are given in blue. As reviewer 3 suggested, we now use a different optimiser to fit our model and ensure our fits are in a minima and not a saddle point. Our "optimal" solution has not changed significantly, but this has impacted the uncertainty around our estimates as previously some refits were saddle points.

Reviewer 1:

OVERVIEW

The authors have addressed the concerns that I raised in my initial review. Before I recommend the manuscript for publication, I would like to raise one point and also request some clarifications from the authors regarding the simulation sweep.

Thank you for your comments. We have addressed your points below.

COMMENTS

In the caption for Figure 2, the authors note that they only plot kernels where all the parameters lie within the respective 95% quantiles. Can the authors justify their reasoning for doing so as opposed to plotting all of the 10,000 inferred parameter sets to show the full range of inferences that they can obtain using the proposed method? It seems to me that restricting the plots to only those parameters sets for which all parameters lie within the respective 95% quantiles would misrepresent the extent to which the inferences reproduce the simulated kernels. I may be misunderstanding this though.

We chose to present kernels where all the parameters lie within the respective 95% confidence intervals because occasionally the unbounded optimiser returned invalid solutions where alpha < 0 and delta < 0. Our initial idea was to show where 95% of the fits lay. However we have now changed Figure 2 to show all solutions, since we have refit our model using a different optimiser. We now find we get solutions in two local regimes - one close to the true value (which corresponds to a lower negative log likelihood) and ones with much larger initial contribution from the background intensity. We show an unmagnified version of Fig2B in Fig S1 - only a few simulations <2% are not shown in the magnified version.

I appreciate the inclusion of a simulation study to examine how the parameter estimates change with the extent of reporting of cases. There did not appear to be a mention of how *Rc* estimates change with the extent of reporting of cases. Because much of the strength of the method is its ability to estimate the reproduction number under control, the authors should mention how this quantity is affected by reporting in their simulation studies.

Thank you for this interesting suggestion. We have now added this extra figure to the supplementary information that shows how the median and 95% confidence intervals of Rc vary with under reporting. We have also added text to the simulation results section showing that our median value decreases.

Moreover, it would be useful if the authors examined the full range of underreporting from 10- 100% to get a sense of at what point inferences break down and therefore where the use of the method may be most

appropriate. Even among near-elimination settings, the quality of surveillance systems varies, so the 70% lower bound of reporting may be overly optimistic in some settings.

Thank you for this suggestion, we have now repeated this analysis to include under-reporting from 10-100% and update Figure 3 accordingly. Please note the results have slightly changed due to our new fitting routine. We find that alpha and delta (the parameter in the kernel) are more robust to under reporting and that, as expected, the terms that control the background intensity or importations (A, B, M and N) change most to account for the secondary infections where the parent case has been removed. We have added an extra sentence to the results to reflect this.

[Lines 372-373] The authors should tone down the claim that being able to forecast case counts five weeks in advance would enable policy makers to take action to reduce transmission. While I agree that forecasts are useful, the method, as presented in this manuscript, lacks a spatial comment. As one of the other reviewers noted, without spatial information to guide these forecasts, it would be challenging to take actionable steps to reduce transmission in light of these forecasts.

Thank you for this comment. We have softened the language to *"This could provide insights to policy makers about short term transmission, which would be further improved by adding in a spatial component."*

Reviewer 2:

I am pleased with your answers to my comments.
Thank you for participating in the peer review of this paper.

Reviewer 3:

I thank the authors for their revisions, which have addressed most of my comments from the first version. As described below, I still have some concerns about the model, primarily about the optimization problems the authors discuss in their reply and about the simulation studies. I think these two problems are connected—better simulation stud- ies may reveal what is going on with the optimization—and the authors should carefully investigate and resolve these issues to ensure confidence in their results.

Thanks for your new comments, we have addressed them below.

Main Comments

1. The authors note in their reply that

We have further investigated the convexity of our negative log-likelihood and found that the eigenvalues of our hessian (returned numerically by the optim solver) do not all have the same sign and so our optimisation finds a saddle point, thus the objective function is not convex.

This makes me suspicious. Does this consistently occur regardless of the starting values used in the optimization? And are these eigenvalues with the wrong sign on the same scale as the other eigenvalues,

or are they, for instance, 10−16? I'm suspicious because (like Reviewer 4) I would be surprised of convergence problems for these models. I'd be extra-surprised if multiple optimization runs from different starting parameter values consistently end up in the same saddle point. I wonder if there is an error in the calculation of the Hessian or of the likelihood, or if the eigenvalues with the wrong sign are very close to zero and actually a result of numerical issues in estimating the Hessian. (In that case, they may indicate non-identifiability in the model, which would be a separate problem to solve.)

In either case, I think this warrants some double-checking (with different starting values and optimizer settings, and of the gradient and likelihood code). A good strategy to diagnose the problem (though perhaps not necessary to present in the paper, depending on what you find) is to make contour plots of the log-likelihood near the solution, varying the parameter with the suspicious eigenvalue and another parameter.

If the saddle point issues are real, they should be mentioned in the text as a warning for the interpretation of the results.

Thank you very much for drawing this strange behaviour to our attention as something we should be concerned about. The optimal solutions from the "optim" that we originally obtained were different so we assumed we were hitting multiple different saddle points. However, we have looked into this more and have implemented a few changes.

1) We compared the numerical hessian from the "optim" package with two other numerical hessian packages from "pracma" and "numDeriv". We found that the "pracma" and "numDeriv" gradients agreed with each other and were different to the ones in the "optim" package. This suggested that optimisers may have difficulty with finding the gradients and hessians of our log-likelihood. We had always been providing an analytic gradient.
2) We derived an analytic hessian and added this to our "epihawkes" package. We found our analytic hessian agreed with the numerical hessians from "pracma" and "numDeriv". This suggested the optimal solutions provided by "optim" were not necessarily optima and we should look for alternative optimisers.
3) We investigated various different open source optimisers and found that "optimx" used the numerical gradients from the "numDeriv" package, which agreed with our analytic hessian. We tried to provide our analytic hessian to the "optimx" package but sometimes the optimiser would throw an unhelpful error message about our hessian potentially being incorrect but nothing else. We check the analytic hessian and the numDeriv hessian at these parameters and find they agree, which agrees with our previous findings that our numerical hessian is derived and coded correctly . We are looking into contacting the developers of that package to suggest a more helpful error message is provided.
4) We therefore have switched to the optimizer in "optimx" and provide our analytic gradients. We check the gradients and hessians at the optimal solutions from 10 different initial positions. We find that the gradients are small for all our optimal solutions. For the China data set, some of our optimal solutions are at saddle points (one negative eigenvalue) but we also find a true minima where all the eigenvalues are positive, see figure A below. For Eswatini we identify two minima regions, where we choose the one with the lower negative log likelihood, see figure B below. We feel this non-convex surface may arise due to the periodic nature of our background intensity. We investigated fitting an exponential and Rayleigh kernel with and without a delay for no background intensity and found our surface was convex.
5) We now ensure for each of our refits that we are not in a saddle point but a true minima.

6) We have updated the text to suggest our solution may be non-convex and that we ensured our optimal solutions were not saddle points. We note our “optimal” solution has not changed, but this refitting alters our uncertainty.

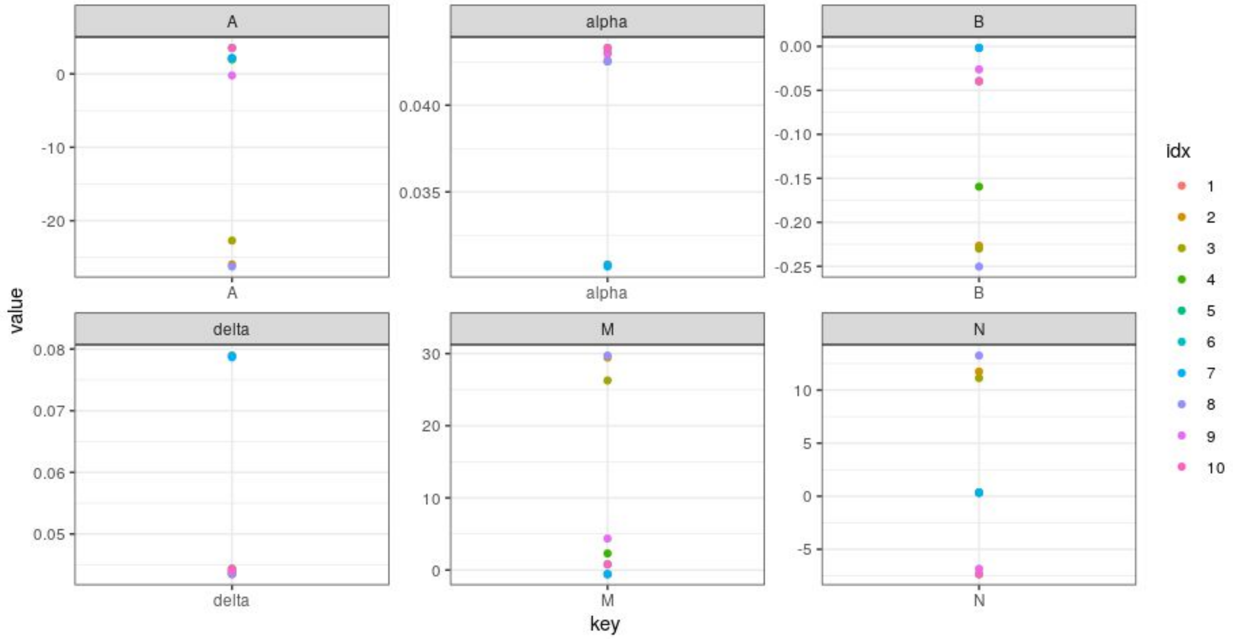

Fig A: Parameters for China dataset

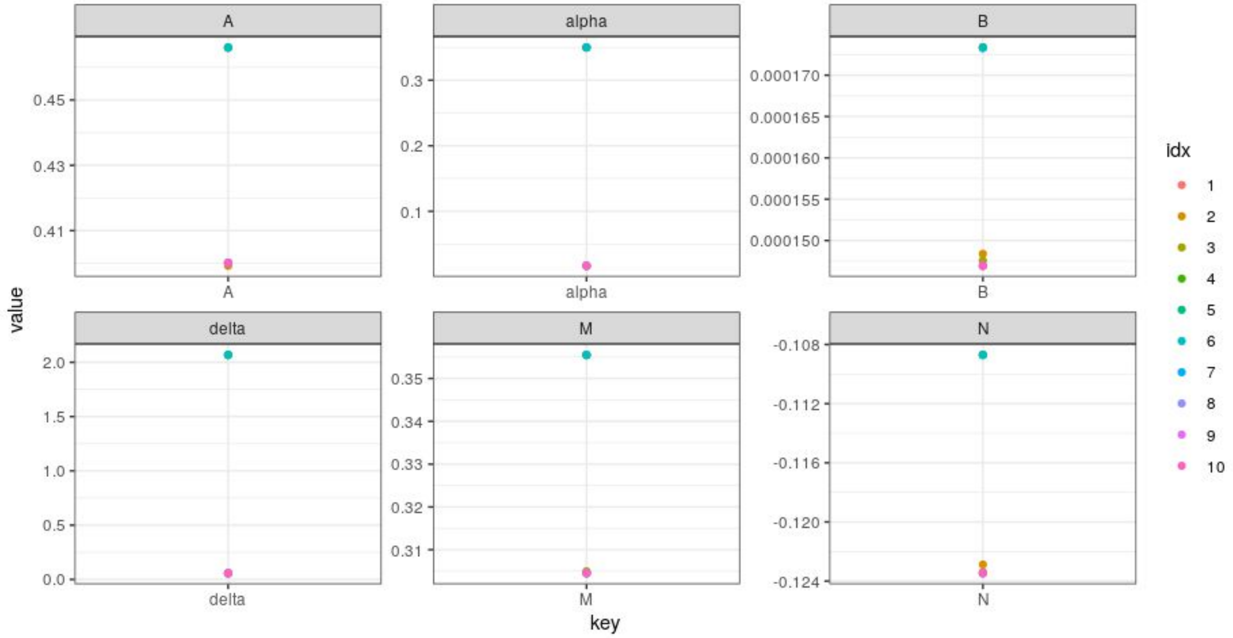

Fig B: Parameters for Eswatini dataset

2. I appreciate that the authors added simulations to validate that their model can re- cover the true parameters. The text (pages 6–7) states that

We simulate 10,000 sets of events... We then use optim to minimise our log-likelihood and find the optimal values of our simulation from one initial set of parameters for each simulation. We compare these fitted parameters to the initial parameters used for the simulation and run KS tests for a sub-sample of our re-fits to check our goodness of fits.

I don’t understand the procedure being described here. I assume “find the optimal values of our simulation from one initial set of parameters for each simulation” simply means the model is fit to the simulated data.

Thanks for pointing out that our method was unclear. We did fit the model to simulated data. We have simplified the text as suggested. It now reads:

“We then use optim to minimise our log-likelihood and find the optimal values of each of our simulations.”

But what are the KS tests testing? The hint is on page 5: “If the model is correct, then, according to the theorem, the difference in intensity between two subsequent events are independent exponential random variables with mean 1.”

This seems to suggest that $\lambda(t_{i+1}) - \lambda(t_i) \sim \text{Exp}(1)$, which is false. (The difference can easily be negative, for instance.) So I hope the text isn’t accurate, and the authors are instead applying the time-rescaling theorem correctly.

We are sorry for the wrong description of the time re-scaling theorem in the text. We have now changed the methods section to reflect what we are actually doing, which is looking at the difference between the integrals of the intensity.

We use the time--rescaling theorem to assess our model fits. Similar to Brown et al. ~\cite{brown:2002}, we define

\begin{equation}

\Lambda(t_{i}) = \int_0^{t_{i}} \lambda(t) \diff t.

\end{equation}

If the model is correct, then, according to the theorem, the difference in Λ, the integral of the intensity, between two subsequent events are independent exponential random variables with mean 1.

3. Also on the topic of the simulation studies, I think what I was really looking for was that the parameters are recovered accurately. If different parameter values can result in similar intensity functions, then interpretation of parameters of the model—like the reproduction number—depends on whether the MLE is estimating the parameters well. And if the MLE is finding a saddle point, as the authors note, that seems questionable. (If, on the other hand, the MLE estimates the parameters quite accurately, that suggests it’s finding the true maximum, not a saddle point, or that the saddle point is somehow always near the maximum. So I think these points are linked: simulations can shed light on what’s going on with the likelihood, and make the implications of the optimization problem clearer.)

The diagnostics I’m thinking of would include, say, a histogram of estimated values of a parameter from the 10,000 simulations, with a line marking the true value. Or a table showing each true parameter value,

plus the mean, median, and quantiles of estimates from the simulation. Either would indicate if the estimation algorithm is indeed working. Reinhart and Greenhouse (2018, Section 4) provide some simulation studies of a related model that illustrate how simulations can be used to illustrate the model's properties.

We agree that presenting results to show if our parameters from the simulation can be accurately recovered. We already include this in the 100% "percentage of data fit to" box in the box and whisker plot, where the red line is the true value. We have edited the text to make this clearer.

4. In my previous review, I suggested plotting the event times $\{ti\}$ against the integral $\int\lambda(t)\,dt$. As the authors correctly note in their reply, this need not be a diagonal 0 line like I implied. What I should have said is that plotting the event indices $\{i\}$ against the integral $\int\lambda(t)dt$ should yield a diagonal line. This, I think, would be a more useful diagnostic than some of the plots included in Figure 2, which don't really demonstrate whether the model is adequately fitting the observed data.

Thanks for your suggestion about Figure 2. We have now included a plot where we plot the integral of our intensity against the event indices for several different simulations.. We find our simulations lie close to a straight line. We also show how the integral of the intensity varies for our two case studies (Figure S2).

5. The authors have moved discussion of the simulation algorithm and $\lambda*$ to the sup- plementary information. I think this is a good choice, but I suspect the paragraph at the bottom of page 4 should be adjusted, because it now mentions "the thinning Algorithm" and "$\lambda*$ is no longer trivial to find" before the algorithm is introduced. $\lambda*$ also no longer seems to be defined in the text.

Thanks for this comment. We have now removed the detail and refer the reader to later in the text:

'This delay is novel and requires modifications to be made to the usual simulation approach; this is explained further below.'

(The same paragraph should also specify the units, days, when stating that $\Delta = 15$.)

We thank you for noticing our omission. We have added this into the manuscript.

Reviewer 4:
The manuscript has greatly improved thanks to the many reviewers' thoughtful comments. The simulation study with its assessment of underreporting is very useful.
We agree with you that thanks to the reviewers comments this manuscript has been improved.

I'm happy with most replies to my comments and only have some minor follow-up remarks:

1. I agree that a purely temporal Hawkes model is a suitable starting point for the development of more complex spatio-temporal formulations such as "twinstim" of [26]. Purely temporal models are much faster to estimate as they don't require heavy cubature over space to evaluate the log-likelihood. FWIW, it is relatively straightforward to supply different parametric kernels in "twinstim" such as the Rayleigh kernel. I'm happy to help if you would like to use "twinstim" for comparison in the future. However, in my experience, reliable estimation of the temporal kernel in a spatio-temporal model requires a lot of events

because the spatial decay reduces the effective number of events contributing to the likelihood. The Eswatini data seem to be too sparse for that, in particular if event locations are partially unknown.

Thanks for agreeing to help with comparisons in the future. We would be interested in this and plan to get in touch after the review has ended.

2. The authors say they now reference Menon and Lee (2018) and Lime and Choi (2018). However, I couldn't find these references and the comment on the negative log-likelihood being potentially non-convex seems gone as well?

We're sorry about this mix up. We must have removed some of the text - following on from Reviewer 3 we have done analysis to investigate the non-convex surface and found the references were no longer relevant. We believe our surface is non-convex and by changing our optimiser to "optimx" instead of "optim", we can distinguish between true minima and saddle points.

3. IMO, Fig S1 would rather suggest that the exponential and Rayleigh kernels fit equally well. I cannot see a relevant difference. Why not report the AIC values to compare the two fits?
Thanks for this suggestion - we have included the AIC in the paper. We find that the AIC for the Rayleigh kernel is slightly smaller than the AIC for the exponential kernel for China (ray - 339.7, exp - 343.5) but that the exponential kernel AIC is slightly lower for Eswatini (ray - 1614, exp - 1606). Due to the biology, we chose the Rayleigh kernel.

Sebastian Meyer
